# Subtypes of cognitive impairment in cerebellar disease identified by cross-diagnostic cluster-analysis: results from a German multicenter study

**DOI:** 10.1007/s00415-024-12831-1

**Published:** 2024-12-21

**Authors:** Qi Liu, Kerstin Rubarth, Jennifer Faber, Patricia Sulzer, Imis Dogan, Miriam Barkhoff, Martina Minnerop, Adam M. Berlijn, Saskia Elben, Heike Jacobi, Julia-Elisabeth Aktories, Dana M. Huvermann, Friedrich Erdlenbruch, Raquel Van der Veen, Johanna Müller, Enzo Nio, Benedikt Frank, Martin Köhrmann, Elke Wondzinski, Mario Siebler, Kathrin Reetz, Jürgen Konczak, Frank Konietschke, Thomas Klockgether, Matthis Synofzik, Sandra Röske, Dagmar Timmann, Andreas Thieme

**Affiliations:** 1 Department of Neurology, Essen University Hospital, University of Duisburg-Essen, Hufelandstraße 55, 45147 Essen, Germany; 2 Center for Translational Neuro- and Behavioral Sciences (C-TNBS), Essen University Hospital, University of Duisburg-Essen, Essen, Germany; 3 Institute of Biometry and Clinical Epidemiology, Charité-University Medicine Berlin, Corporate Member of Freie University, Berlin, Germany; 4 German Center for Neurodegenerative Diseases (DZNE) Bonn, Bonn, Germany; 5 Department of Neurology, Bonn University Hospital, Rheinische Friedrich-Wilhelms University, Bonn, Germany; 6 Division Translational Genomics of Neurodegenerative Diseases, Hertie Institute for Clinical Brain Research and Center of Neurology, Eberhard-Karls University Tübingen, Tübingen, Germany; 7 German Center for Neurodegenerative Diseases (DZNE) Tübingen, Helmholtz Association, Tübingen, Germany; 8 Department of Neurology, University Hospital RWTH Aachen, Rheinisch-Westfälische Technische Hochschule (RWTH) Aachen, Aachen, Germany; 9 JARA-BRAIN Institute Molecular Neuroscience and Neuroimaging, Research Center Jülich GmbH, Jülich, Germany; 10 Department of Neurology, Center for Movement Disorders and Neuromodulation, Medical Faculty and University Hospital Düsseldorf, Heinrich-Heine University Düsseldorf, Düsseldorf, Germany; 11 Institute of Clinical Neuroscience and Medical Psychology, Medical Faculty and University Hospital Düsseldorf, Heinrich-Heine University Düsseldorf, Düsseldorf, Germany; 12 Institute of Neuroscience and Medicine (INM-1), Research Center Jülich GmbH, Jülich, Germany; 13 Department of Neurology, Heidelberg University Hospital, Ruprecht-Karls University, Heidelberg, Germany; 14 Faculty of Mathematics and Natural Sciences, Heinrich-Heine University Düsseldorf, Düsseldorf, Germany; 15 Department of Neurology and Neurorehabilitation, MediClin Rhein/Ruhr, Essen, Germany; 16 Human Sensorimotor Control Laboratory, School of Kinesiology and Center for Clinical Movement Science, University of Minnesota, Minneapolis, USA; 17 Berlin Institute of Health (BIH), Berlin, Germany

**Keywords:** Cerebellar cognitive affective syndrome (CCAS), Subgroups of CCAS, Cerebellar disorders, Cluster analysis, German CCAS-Scale

## Abstract

**Background:**

Cognitive and neuropsychiatric impairment, known as cerebellar cognitive affective syndrome (CCAS), may be present in cerebellar disorders. This study identified distinct CCAS subtypes in cerebellar patients using cluster analysis.

**Methods:**

The German CCAS-Scale (G-CCAS-S), a brief screening test for CCAS, was assessed in 205 cerebellar patients and 200 healthy controls. K-means cluster analysis was applied to G-CCAS-S data to identify cognitive clusters in patients. Demographic and clinical variables were used to characterize the clusters. Multiple linear regression quantified their relative contribution to cognitive performance. The ability of the G-CCAS-S to correctly distinguish between patients and controls was compared across the clusters.

**Results:**

Two clusters explained the variance of cognitive performance in patients’ best. Cluster 1 (30%) exhibited severe impairment. Cluster 2 (70%) displayed milder dysfunction and overlapped substantially with that of healthy controls. Cluster 1 patients were on average older, less educated, showed more severe ataxia and more extracerebellar involvement than cluster 2 patients. The cluster assignment predicted cognitive performance even after adjusting for all other covariates. The G-CCAS-S demonstrated good discriminative ability for cluster 1, but not for cluster 2.

**Conclusions:**

The variance of cognitive impairment in cerebellar disorders is best explained by one severely affected and one mildly affected cluster. Cognitive performance is not only predicted by demographic/clinical characteristics, but also by cluster assignment itself. This indicates that factors that have not been captured in this study likely have effects on cognitive cerebellar functions. Moreover, the CCAS-S appears to have a relative weakness in identifying patients with only mild cognitive deficits.

**Study registration:**

The study has prospectively been registered at the German Clinical Study Register (https://www.drks.de; DRKS-ID: DRKS00016854).

**Supplementary Information:**

The online version contains supplementary material available at 10.1007/s00415-024-12831-1.

## Introduction

Traditionally cerebellar disorders are known to cause motor impairments, known as cerebellar ataxia. In recent years, increasing evidence has shown that patients with cerebellar disorders may also experience cognitive dysfunction [65]. The latter can range from mild difficulties to more severe deficits that significantly impact daily life [13, 49, 56]. Commonly impaired cognitive domains in cerebellar patients include executive, linguistic and visuospatial functions. Moreover, behavioral abnormalities are observed which include disturbances of emotional control and social skill set as well as autism- or psychosis-like behaviors [55]. This complex of non-motor dysfunction is referred to as cerebellar cognitive affective syndrome (CCAS) [54].

For many years, cognitive dysfunction in cerebellar patients was assessed by the use of different neuropsychological test batteries, making a direct comparison between studies difficult [1, 43]. Moreover, given the rareness of primarily cerebellar disorders, most studies had quite small sample sizes [1, 43, 81]. These drawbacks were overcome in part after Hoche and colleagues introduced the *CCAS-Scale* (CCAS-S) which is a brief bedside test to screen for CCAS in patients with cerebellar disorders [20]. The CCAS-S is now in widespread use and makes cognitive screening of cerebellar patients easier, faster and more standardized—allowing for multicenter studies with larger sample sizes and enabling comparison of test results between sites and studies [67].

Although the introduction of the CCAS-S has led to an increase of research regarding the CCAS, no study has yet addressed the exact cognitive profile of distinct subtypes of CCAS. Such subtypes are likely to exist, given the different nature of specific hereditary, sporadic and acquired (symptomatic) ataxias [34]. To date, it is unclear why some patients have more prominent cognitive dysfunction and if different cognitive domains are more likely to be affected in patients with mild or more prominent cognitive impairments. For the overall concept of CCAS, research suggests that the severity of cognitive deficits depends on lesion localization in cerebellar stroke or the pattern of atrophy in degenerative disorders [28, 63] as well as the stage of the disease [15, 59]. Some studies have found a correlation between the severity of motor symptoms (i.e. ataxia) and cognitive deficits [36, 42], while others have not or found mixed results [59, 66]. Further studies have identified factors that influence test results of the now widely used CCAS-S itself, such as age and level of education [51, 59, 68].

To date, the studies examining CCAS by use of the CCAS-S have either included only patients without a healthy control group for comparison [42, 62] or they have utilized a group average approach to compare cerebellar patients with healthy controls [20, 36, 66]. Given the existing age and education effects on CCAS-S performance [51, 59, 68], however, a healthy reference group is needed to correctly interpret CCAS-S test results. The group average approach, on the other hand, often overlooks individual variability within groups and might miss (subtle) patterns present in a dataset [51, 59, 68].

Cluster analysis is a method which overcomes the drawbacks of the conventional group average approach. It is a type of unsupervised machine learning technique which is used to identify distinct clusters (subgroups) in a heterogeneous study sample. Cluster analysis enables to identify submerged patterns within a dataset and the contribution of covariates to these patterns [6, 83]. The method has already been used to analyze cognitive performance measured by other psychometric tests, e.g., designed to assess cognition in patients with schizophrenia or obsessive–compulsive disorders [5, 26, 38]. Although numerous studies have examined cognitive impairments in cerebellar disorders, no study has yet used cluster analysis for this purpose.

Hence, the aim of this study was to identify clusters of patients which share a similar cognitive profile as assessed with the German version of Cerebellar Cognitive Affective Syndrome Scale (G-CCAS-S) [67]. Specifically, we were interested both in the severity of CCAS shared within a cluster of patients and the pattern of the affected cognitive domains. Moreover, we aimed to determine the covariates contributing to these distinct clusters. Finally, we aimed to evaluate the differential ability of the G-CCAS-S to identify patients in the found clusters and we intended to compare the cognitive profiles present in the clusters with that of healthy controls.

## Methods

### Participants

The patients diagnosed with hereditary or sporadic ataxias [e.g., spinocerebellar ataxias (SCA), Friedreich’s ataxia (FRDA), sporadic adult onset ataxia of unknown etiology, multisystem atrophy—cerebellar type (MSA-C) etc.] or acquired cerebellar disease (that is: cerebellar stroke) were recruited at the University Hospitals of Aachen, Bonn, Düsseldorf, Essen, Heidelberg, and Tübingen in Germany (see supplementary material 1, Table S1 for details). The inclusion and exclusion criteria are shown in Table 1, following the same standards as in our previous studies [66–68]. All patients were part of the validation study of the German version of the CCAS-Scale [69].

**Table 1:** Inclusion and exclusion criteria for cerebellar patients and healthy controls

Group	Inclusion criteria	Exclusion criteria
General	18 years or older Fluent German-speaker Informed consent	Alcohol or drug abuse Presence of severe systemic diseases (consuming diseases or poor general health condition) Primary psychiatric disorders or neurological disorders (exception: ataxia in patients) Intake of medicines that can influence the central nervous system (exception: patients on stable dose of antidepressants)
Patients	General inclusion criteria Existence of a degenerative cerebellar disorder or an acquired cerebellar lesion	General exclusion criteria
Controls	General inclusion criteria	General exclusion criteria

The study has been registered prospectively at the German Clinical Study Register (https://www.drks.de; DRKS-ID: DRKS00016854). The study obtained approval from the local ethics committees of all participating sites. All participants gave their written informed consent in accordance with the declaration of Helsinki.

### Demographic characteristics and clinical assessment

Demographics including sex, age, and educational level (measured by number of educational years: primary + secondary + tertiary education) were recorded. The clinical information obtained included the diagnosis and disease duration (that is: time interval between onset of gait ataxia and study inclusion).

Because many hereditary (e.g. SCA1, SCA2, SCA3, etc.) and sporadic (e.g. MSA-C) ataxias also affect other parts of the central nervous (e.g. basal ganglia, cerebral cortex, etc.) which are involved in cognition [30, 31], the degree of extracerebellar involvement is likely to influence cognitive functions [51, 59, 66]. To investigate the influence of extracerebellar involvement on cluster classification, all patients were divided into two subgroups—“cerebellar pure disease” and “cerebellar plus disease”. Subsequently, the distribution of “cerebellar pure disease” and “cerebellar plus disease” across the clusters was registered. The disorders that are considered to have a relatively pure cerebellar pathology, like SCA6, SCA27B, episodic ataxias, isolated cerebellar stroke, etc. were classified as “cerebellar pure disease”. In contrast, disorders with a more complex cerebro-cerebellar pathology, such as SCA1, SCA2, SCA3, Friedreich’s ataxia (FRDA), MSA-C, etc. were classified as “cerebellar plus disease” (supplementary material 1, Table S1) [67, 80].

In 167 patients, the individual extracerebellar involvement was quantified using the Inventory of Non-Ataxia Signs (INAS). The presence of extracerebellar signs and symptoms (such as hyperreflexia, rigidity, dystonia, etc.) is registered by the INAS and adds up to the INAS count (with an INAS count of 0 reflecting no extracerebellar involvement and an INAS count of 16 reflecting a maximum of extracerebellar involvement) [22].

In addition, we evaluated the severity of cerebellar ataxia by use of the Scale for the Assessment and Rating of Ataxia (SARA) in 202 patients. The rating elements within the SARA include gait, stance, sitting, speech, finger chase, nose-finger-test, fast alternating hand movements, and heel-shin slide. The total SARA score varies from 0 to 40, with a higher score reflecting more severe ataxia [57].

### Cognitive assessment

The German version A (versions B-D exist as well) [67] of the Cerebellar Cognitive Affective Syndrome Scale (G-CCAS-S) [20] was used to evaluate cognitive function in cerebellar patients. The G-CCAS-S contains ten test items. Nine items capture deficits in at least one of the four domains of the CCAS (executive, linguistic, visuospatial, and neuropsychiatric functions), and one item assesses episodic memory. Memory impairment is considered as an indicator for extracerebellar involvement in cognitively impaired patients since (as described above) some hereditary and sporadic ataxias are not restricted to the cerebellum [20].

According to Hoche et al. [20] and Thieme et al. [67], for each of the ten test items a raw score was given. Moreover, each item was rated as passed or failed. An item was failed if the item-specific raw score cut-off value was not reached. According to the original US-American validation study by Hoche et al., the patients were considered to have a *possible* CCAS if one item was failed, a *probable* CCAS if two items were failed, and a *definite* CCAS if three or more items were failed [20].

However, these evaluation criteria have yielded a high rate of false-positive results in healthy controls questioning the meaningfulness of these criteria [7, 12, 51, 66]. Our group has found that CCAS-S test performance depends on age and education and to a lesser degree on sex [68, 69]. Therefore, we have recently developed a correction formula [69]. This formula controls for sex, age, and education effects, and indicates whether or not a test result in a patient of a given sex, age, and education is considered cognitively impaired compared to a healthy control with the same demographic characteristics (see supplementary material 1, S1 for details). In this study, we have used both the evaluation approach by Hoche et al. [20] and the approach by Thieme et al. [69] to evaluate sensitivity and selectivity of the CCAS-S in the found clusters.

For the cluster analysis, all G-CCAS-S test items were assigned to the cognitive domains that they capture. Some cognitive domains were captured by only one test item: visuospatial function (corresponding item: cube draw/copy), neuropsychiatric abnormalities (corresponding item: affect) and episodic memory (corresponding item: verbal recall). Other cognitive domains were assessed by more than one item: linguistic functions (corresponding items: semantic fluency, phonematic fluency, category switching), executive functions (corresponding items: semantic fluency, phonematic fluency, category switching, digit span forward, digit span backward, similarities, go/no-go) [2, 77].

### Statistical analysis

#### Cluster analysis and principal component analysis (PCA)

Cluster analysis was performed with R studio (version 2023.09.1 + 494, http://www.rstudio.com/) [64] using the “Cluster package” [37]. The K-means algorithm was utilized which is an iterative clustering method [17, 48]. The *z*-score for each cognitive domain (executive, linguistic, visuospatial, and neuropsychiatric functions as well as episodic memory) was entered into the cluster analysis. The Hopkins index (range: 0 to 1) was used to measure the clustering tendency within the dataset, with values close to 1 indicating a high clustering tendency and those close to 0 reflecting a random distribution [21]. If the database is suitable for cluster analysis, the optimal number of clusters is determined using the elbow plot and the silhouette metric. The silhouette metric (range: − 1 to + 1) was used to assess how similar a data point is to its own cluster (cohesion) and how well it separates from other clusters (separation). A value of + 1 indicates that a data point is perfectly clustered and far away from neighboring clusters, and an index of 0 indicates that a data point is on or very close to the decision boundary between two neighboring clusters. The negative values indicate that data points might be in the wrong cluster [52].

Principal component analysis (PCA), a linear method utilized for data dimensionality reduction by transforming it into a newly coordinated system [23], was implemented based on the *z*-scores for the five cognitive/neuropsychiatric domains. Both patients in clusters and the healthy control subjects were analyzed separately using PCA to illustrate the position of healthy individuals in comparison to the two clusters identified within the cerebellar patients.

#### Cognitive profile within the clusters

Following cluster analysis, the affected domains were compared across clusters to examine if some cognitive domains were more likely to be impaired in one of the clusters. The differences among the clusters and healthy controls in impaired domains, demographic, and clinical characteristics were analyzed using Mann–Whitney *U* tests and one-way analysis of variance (ANOVA) with Bonferroni-corrected post hoc *t*-tests for continuous variables, and Chi-squared tests for nominal variables.

An additional analysis of covariance (ANCOVA) was conducted to investigate the difference of cognitive patterns among clusters while accounting for the effects of potential confounding variables (age and education) [41].

#### Discriminative ability of the G-CCAS-S within the clusters

Next, the sensitivity (that is the ability of the G-CCAS-S to correctly identify patients in the two clusters as patients) was assessed (1) using the classification criteria introduced by Hoche et al. [20], (2) the correction formula by Thieme et al. [69], and (3) the area under (AUC) the receiver operating curves (ROC) as well as the Youden Index (YI)—both for the total sum raw score and for the number of failed test items. For the ROC analysis, we divided the healthy control group into two subgroups, ensuring that each subgroup was matched for age and education within their respective comparison group of patients (cluster 1 or cluster 2). An AUC of smaller than 0.5 indicates that a test does not exceed chance level in discriminating patients from controls, while an AUC of 1 indicates that a test has perfect discriminatory abilities [71]. Consistent with previous studies, AUC scores from 0.5 to 0.7 indicate poor, 0.7 to 0.8 imply good, and AUC scores above 0.8 reflect excellent discriminative abilities [11, 16]. YI was utilized to calculate the optimal cut-offs (that is the optimal balance between true positives and false negatives) for total failed items and total sum raw score (YI = sensitivity + selectivity—1 for a specific cut-off) [7, 82].

#### Regression analyses

A series of multiple linear regression models was conducted to examine whether cluster assignment explained cognitive performance beyond the other aforementioned covariates. According to prior research, sex, age, education, disease duration, diagnosis, and the performance of SARA and INAS were selected as the covariates of interest [51, 59, 66, 68]. The categorical variables (sex and diagnosis) were included in the regression model by using dummy coding.

In all statistical tests, *p*-values < 0.05 were considered to be statistically significant (2-sided). The statistical analyses were conducted using JASP (version 0.18.3) and R studio (version 2023.09.1 + 494), and the figures were created with R studio by using the packages “ggplot2” [78], “factoextra” [27] and “dplyr” [79].

## Results

### Demographic and clinical characteristics

A total of 205 patients with cerebellar disorders and 200 healthy controls was included in this study. Within the patient group, 87 individuals were classified into the “cerebellar pure disease” subgroup, while 118 were assigned to the “cerebellar plus disease” subgroup. A total of 30 forms of ataxia was included in the study. The most common types of ataxia were SCA3 (*N* = 44), FRDA (*N* = 22), SCA6 (*N* = 19), SCA14 (*N* = 12) and ataxia due to cerebellar stroke within the territory of the posterior inferior cerebellar artery (PICA stroke; *N* = 17, whereof 15 unilateral, and 2 bilateral).

The mean age and educational level were not significantly different between the group of all patients (age: 53.2 ± 14.6 years; education: 14.9 ± 3.2 years) and healthy controls (age: 51.4 ± 18.1 years; education: 15.4 ± 2.8 years). However, the sex distribution differed between patients and controls (male patients: 56% vs. male controls: 46%, *p* = 0.042, Chi-squared test). Table S2 (in supplementary material 1) provides a comparison of demographic characteristics for the entire group of patients and healthy controls while Tables 3 gives more clinical information for the patients within each cluster.

### Clusters of cognitive dysfunction in cerebellar patients

**Fig. 1:**
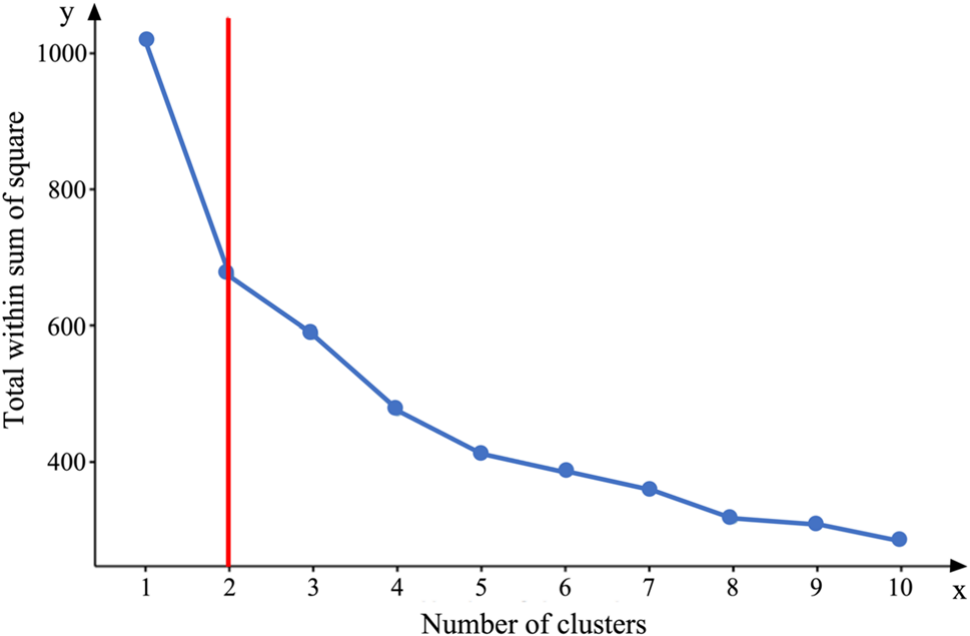
**K-mean cluster elbow graph.** The abscissa (*x* = 2, red line) of the inflection point in the K-means cluster elbow graph indicates the most appropriate number (2) of clusters

**Fig. 2:**
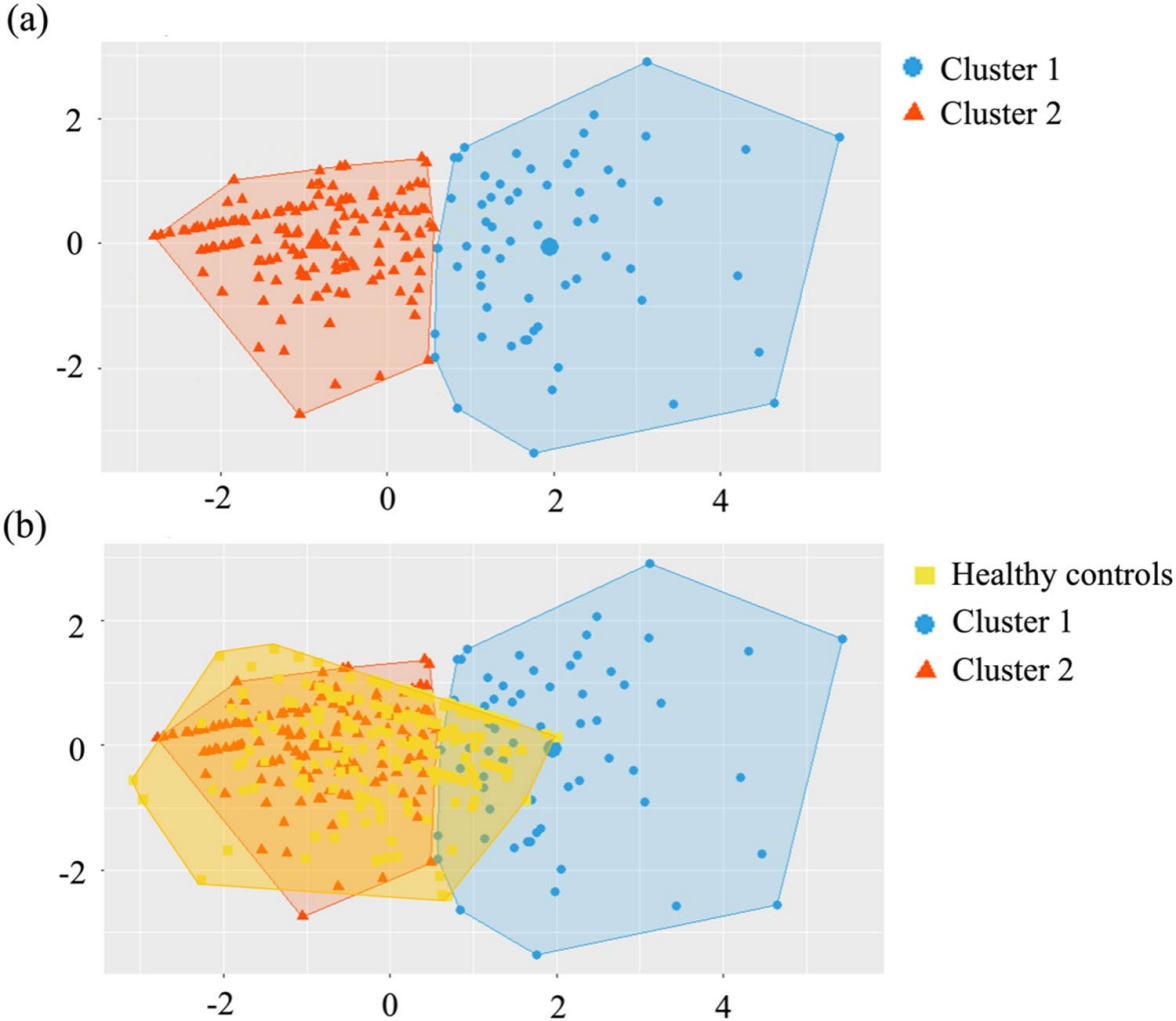
**Relation of clusters to each other and to healthy controls.** **a** The two distinct clusters of patients are shown in comparison to each other. Patients with mild cognitive impairment are shown on the left (cluster 2, red), patients with severe cognitive impairment are shown on the right (cluster 1, blue). **b** The two clusters of patients are shown in comparison to the cluster of healthy controls (yellow). A substantial overlap between patients in cluster 2 and healthy controls is visible. The results from the principal component analysis revealed that principal component 1 (dimension 1, *x* axis) accounts for 50% of the variance in the original data, while principal component 2 (dimension 2, *y* axis) accounts for 18% of the variance in the original data

**Fig. 3:**
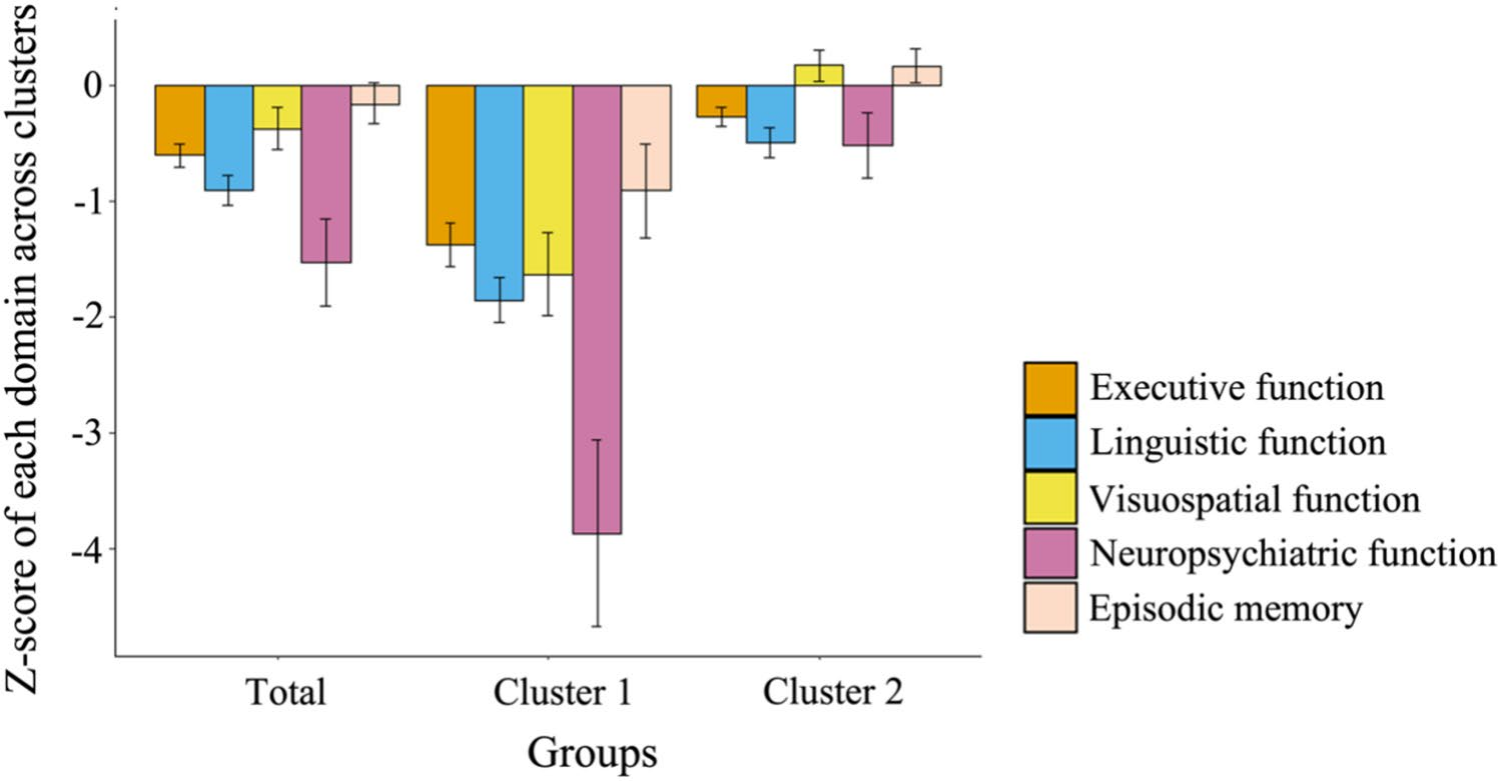
**Z-scores** **of** **cognitive/neuropsychiatric** **domains** **across** **clusters.** The figure shows the mean *z*-score for each cognitive and neuropsychiatric function for the total group and for the two clusters of cerebellar patients

The Hopkins index was 0.79 which indicated the strong clustering tendency of the dataset. A Hopkins index close to + 1 indicates both a good cohesion within each cluster and good separation between the two clusters. As depicted in Fig. 1, the maximal inflection point in the elbow graph supported the choice of two clusters. In addition, the average silhouette width for the two-cluster solution was 0.38, which was the highest value, and therefore, also favors the two-cluster solution (supplementary material 1, Table S3). In the cohort of patients with cerebellar disorders, 62 individuals (30%) were classified into cluster 1, while 143 (70%) were attributed to cluster 2. Following the cluster analysis, a PCA was performed among patients in the two clusters and the healthy control group to visualize the distribution of cognitive performance of patients in cluster 1 and cluster 2 in relation to healthy controls (Table 2, Figs. 2, 3 and Table S4 in supplementary material 1). This demonstrated a substantial overlap between patients in cluster 2 and healthy controls, whereas there was only a minimal overlap observed between patients in cluster 1 and healthy controls (Fig. 2).

**Table 2:** **Cognitive/neuropsychiatric domains in clusters**

	Healthy controls *n* = 200	Cluster 1 *n* = 62	Cluster 2 *n* = 143	Effect size ^a^	*p* value
Executive function	0.00 ± 0.51	− 1.38 ± 0.73	− 0.27 ± 0.50	0.43	< 0.001^b, c, d^
Linguistic function	0.00 ± 0.74	− 1.86 ± 0.75	− 0.49 ± 0.79	0.41	< 0.001^b,c,d^
Visuospatial function	0.00 ± 1.00	− 1.63 ± 1.42	0.18 ± 0.83	0.27	< 0.001^b,c^
Neuropsychiatric function	0.00 ± 1.00	− 3.87 ± 3.19	− 0.52 ± 1.74	0.36	< 0.001^b,c,d^
Episodic memory	0.00 ± 1.00	− 0.91 ± 1.61	0.17 ± 0.92	0.10	< 0.001^b,c^

Statistics: one-way analysis of variance (ANOVA) followed by Bonferroni-corrected post hoc *t*-tests were used for continuous variables

^a^*ω*^2^ was used to evaluate the effect size

^b^Significant difference between healthy controls and cluster 1 (*p* < 0.05)

^c^Significant difference between cluster 1 and cluster 2 (*p* < 0.05)

^d^Significant difference between healthy controls and cluster 2 (*p* < 0.05)

### Cognitive profile within clusters

Statistically significant differences were observed between the two clusters of patients and healthy controls across all G-CCAS-S test items and across all domains (all *p* < 0.001, ANOVA; Table 2 and Table S4 in supplementary material 1). In particular, post-hoc analyses illustrated that cluster 1 had significantly lower mean *z*-scores in all domains compared to cluster 2 and healthy controls (all *p* < 0.001, Bonferroni-corrected post hoc *t*-tests). Moreover, in comparison to healthy controls, cluster 2 demonstrated significantly worse performance within the executive (*p* < 0.001), linguistic (*p* < 0.001), and neuropsychiatric domain (*p* = 0.023). However, no significant differences were observed in visuospatial functions and episodic memory between patients in cluster 2 and healthy controls. Considering the significant differences in age and educational level among the clusters and healthy controls (Table 3), we conducted an ANCOVA analysis with these variables as covariates to adjust for their effects. The outcomes were found to be in line with the unadjusted results (Table S5 in supplementary material 1).

**Table 3:** **Demographic and clinical characteristics of patients in clusters**

	All patients *n* = 205	Cluster 1 *n* = 62	Cluster 2 *n* = 143	Effect size^a^	*p* value
Age (years)	53.2 ± 14.6	57.4 ± 13.4	51.4 ± 14.7	0.24	0.006
Education (years)	14.9 ± 3.2	13.2 ± 2.7	15.6 ± 3.1	0.43	< 0.001
Male, *n* (%)	115 (56%)	33 (53%)	82 (57%)	0.04	0.585
Cerebellar plus disease, *n* (%)	118 (58%)	37 (60%)	81 (57%)	0.03^e^	0.686^e^
Cerebellar pure disease, *n* (%)	87 (42%)	25 (40%)	62 (43%)		
Disease duration (years)^b^	13.4 ± 11.3	14.5 ± 12.6	12.9 ± 10.7	0.08	0.377
SARA score^c^	12.4 ± 7.8	14.9 ± 7.7	11.3 ± 7.6	0.29	0.001
INAS count^d^	3.2 ± 2.4	3.7 ± 2.1	2.9 ± 2.4	0.27	0.005

Statistics: Mann–Whitney *U* tests were used for continuous variables and Chi-squared tests for classification variables

*SARA* Scale for the Assessment and Rating of Ataxia, *INAS* Inventory of Non-Ataxia Signs

^a^The effect size was evaluated using the rank biserial correlation for continuous variables and Cramer’s *V* index for categorical variables (sex and diagnoses)

^b^Missing data: one in cluster 2

^c^Missing data: one in cluster 1 and two in cluster 2

^d^Missing data: eight in cluster 1 and thirty in cluster 2

^e^Distribution of cerebellar pure disease and cerebellar plus disease across clusters

**Fig. 4:**
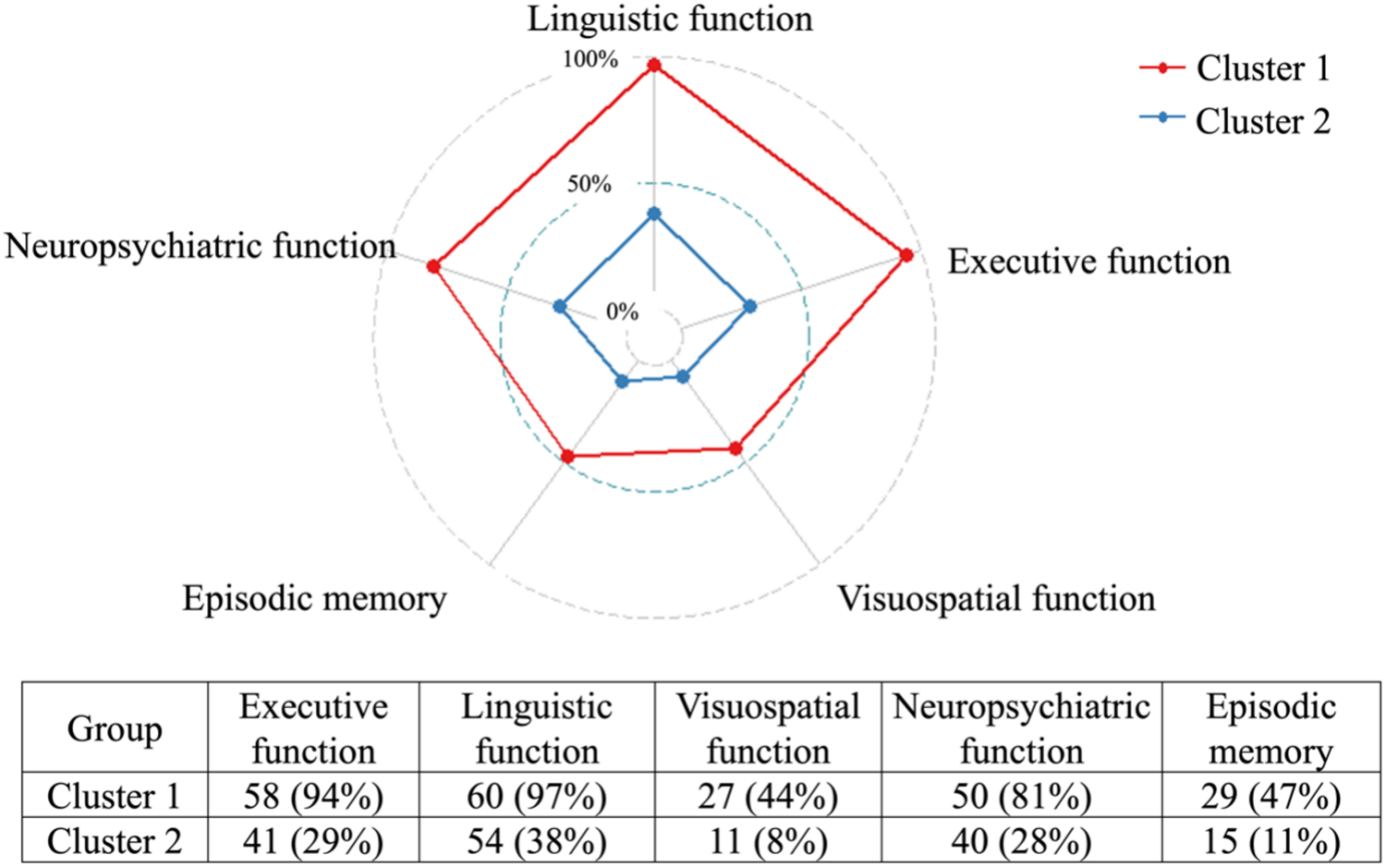
**Percentage of patients impaired in each cognitive and the neuropsychiatric domain across clusters.** The absolute number and the percentage (in parentheses) of patients with deficits in the four core domains of CCAS as well as the memory domain (which is not part of CCAS, but rather indicates cerebral involvement) is shown. A patient was considered impaired on a certain domain if his/her *z*-score was one standard deviation or more below the healthy control group’s average

The percentage of patients in each cluster experiencing impairment on the single cognitive domains is shown in Fig. 4 The most impaired domains were linguistic (cluster 1: 97% vs. cluster 2: 38%), executive (cluster 1: 94% vs. cluster 2: 29%) and neuropsychiatric functions (cluster 1: 81% vs. cluster 2: 28%). Visuospatial functions (cluster 1: 44% vs. cluster 2: 8%) and episodic memory (cluster 1: 47% vs. cluster 2: 11%) were less affected. Note, that the latter is not part of the CCAS, but rather indicates cerebral involvement which may be present in some hereditary or sporadic ataxias.

### Diagnostic accuracy of the G-CCAS-S within the clusters

To evaluate the discriminative ability of the G-CCAS-S, different methods were utilized. First, we used the classification criteria for interpretation of CCAS-S results introduced by Hoche et al. [20]. According to these, 21/19/47% met the criteria for possible/probable/definite CCAS respectively in the entire group of cerebellar patients. In comparison, the percentages were 33/22/18% in the healthy control group. Within the clusters, the distribution for CCAS possible/probable/definite were 0/3/97% for cluster 1 and 26/26/30% for cluster 2.

Next, we applied the correction formula developed by Thieme et al. [69] controlling for sex, age and education effects. According to this approach, 73% of the patients showed cognitive impairment in the whole group of the patients. In cluster 1, 97% and in cluster 2, 62% were classified as “cognitively impaired” (Figs. S1, S2).

**Fig. 5:**
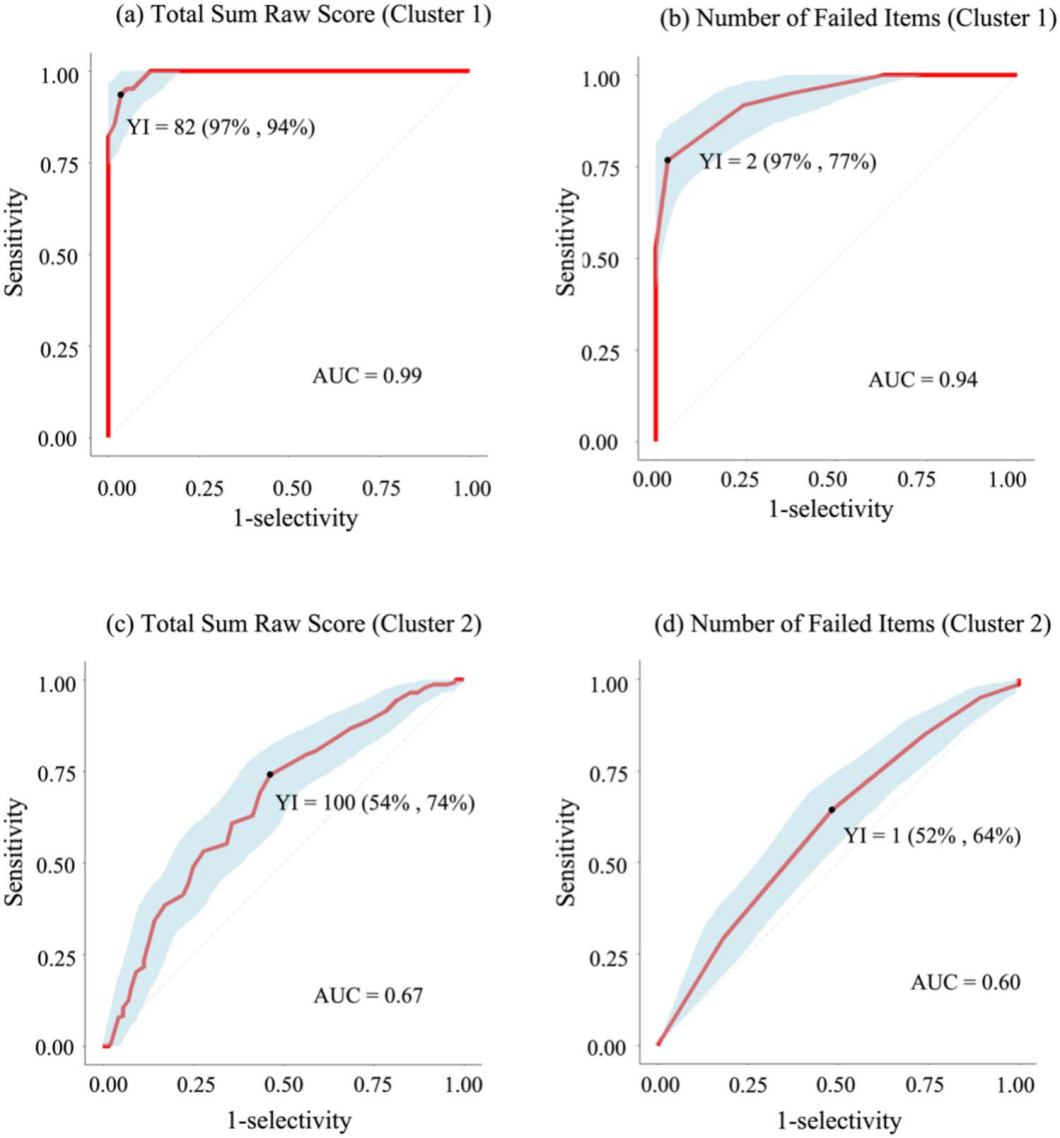
**Ability of the G-CCAS-S to differentiate patients within each cluster from age- and education-matched healthy controls.** ROC curves (red line) of G-CCAS-S are shown for the total sum raw score of cluster 1 (**a**) and cluster 2 (**c**) as well as for the number failed items of cluster 1 (**b**) and cluster 2 (**d**). The 95% confidence interval for each ROC curve is also displayed in the figure (depicted by blue shadow). For each measure, the Youden Index (YI, black dot) is shown and the values for selectivity (that is: the portion of controls correctly identified as controls/all controls in the respective control group) and sensitivity (that is: the portion of patients correctly identified as patients/all patients in the cluster) for the respective YI are given in parentheses. *G-CCAS-S* German version of Cerebellar Cognitive Affective Syndrome Scale, *ROC* receiver operating characteristic, *YI* Youden Index, *AUC* area under curve

Finally, we used the area under the curve (AUC) and the receiver operating curves (ROC) to evaluate the discriminate ability of the G-CCAS-S for each of the two identified clusters of patients in comparison to an age- and education-matched healthy control group respectively (Tables S6, S7 in supplementary material 1). For differentiation between patients in cluster 1 and healthy controls, the AUC was 0.99 (95% confidence interval (CI): 0.98–1.00) for the G-CCAS-S total sum raw score and 0.94 (CI 0.90–0.98) for the number of failed items (Fig. 5). For the differentiation between patients in cluster 2 and healthy controls, the AUC for the total sum raw score and the number of failed items were 0.67 (CI 0.60–0.73) and 0.60 (CI 0.54–0.66) respectively (Fig. 6).

Using the YI, the ideal cutoff value of the G-CCAS-S total sum raw score was 82 points, yielding a sensitivity of 94% and a selectivity of 97% to differentiate patients in cluster 1 from healthy controls. The same approach identified an optimal cutoff for total sum raw score of 100 points with a sensitivity of 74% and a selectivity of 54% for cluster 2. For the number of failed test items, the ideal cutoff for cluster 1 was 2 failed items (sensitivity: 77%, selectivity: 97%), and 1 failed item for cluster 2 (sensitivity: 64%, selectivity: 52%; Fig. 5).

### Demographic and clinical characteristic of the clusters

The patients in cluster 1 were significantly older (*p* = 0.006, Mann–Whitney *U* test) and had a lower level of education (*p* < 0.001) than those in cluster 2. Both groups did not differ regarding sex (*p* = 0.585, Chi-squared test; Table 3).

In terms of clinical features, the patients in cluster 1 suffered from more severe ataxia, as measured by the SARA (*p* = 0.001, Mann–Whitney *U* test) and they had a larger burden of non-ataxia signs, as evaluated by the INAS (*p* = 0.005) than those in cluster 2. The average disease duration did not differ between the two clusters (Table 3).

To examine, if patients with a specific diagnosis were more likely to fall into one of the two clusters, the distribution of diagnoses between the two clusters was compared. The categorization as “cerebellar pure disease” and “cerebellar plus disease” was evenly distributed across the two clusters (*χ*^2^ = 0.2, *p* = 0.686, Chi-squared test; Table 3). For a detailed distribution of each disease across clusters, please refer to Table S1 (supplementary material 1).

### Linear regression

To validate if the cluster attribution explains performance on the single cognitive domains even after adjusting for demographic (age, education and sex) and clinical (disease duration, SARA score, INAS count, diagnosis) characteristics, a series of multiple linear regression analyses was conducted. The models with these predictors accounted for a significant amount of variance in all domains (see Table S8 in supplementary material 1 for details).

As indicated in Table 4, cluster attribution contributed to all the models for domains even after accounting for the other variables [all *t* (8) ≥ 4.75, all *p* < 0.001, linear regression]. Age was a significant predictor of performance in the episodic memory [*t* (8) = − 4.07, *p* < 0.001] and the neuropsychiatric domain [*t* (8) = 2.47, *p* = 0.014], while education only significantly contributed to executive [*t* (8) = 3.58, *p* < 0.001] and linguistic functions [*t* (8) = 2.07,* p* = 0.041]. The males performed better on visuospatial function [*t* (8) = − 2.08,* p* = 0.039] and females performed better on linguistic function [*t* (8) = 2.22,* p* = 0.028]. Disease duration was a significant predictor for visuospatial functions [*t* (8) = − 2.40,* p* = 0.017]. Diagnosis and the SARA score were not significant predictors in any model.

**Table 4:** **Linear regression coefficients**

	*b*-value ± SE	*t*-value	95% CI (lower bound, upper bound)	*p* value
**Executive function**				
Age (years)	− 0.01 ± 0.00	− 1.82	− 0.01, 0.00	0.071
Education (years)	0.06 ± 0.02	3.58	0.03, 0.09	< **0.001**
Sex	0.11 ± 0.09	1.23	− 0.07, 0.29	0.220
Disease duration (years)	− 0.00 ± 0.00	− 0.10	− 0.01, 0.01	0.923
SARA score	− 0.01 ± 0.01	− 0.63	− 0.02, 0.01	0.530
INAS count	− 0.02 ± 0.03	− 0.79	− 0.07, 0.03	0.433
Diagnosis	0.06 ± 0.11	0.56	− 0.15, 0.27	0.578
Cluster	0.88 ± 0.10	8.54	0.67, 1.08	< **0.001**
**Linguistic function**				
Age (years)	− 0.01 ± 0.00	− 1.08	− 0.01, 0.00	0.280
Education (years)	0.04 ± 0.02	2.07	0.00, 0.08	**0.041**
Sex	0.26 ± 0.12	2.22	0.03, 0.50	**0.028**
Disease duration (years)	0.00 ± 0.01	0.02	− 0.01, 0.01	0.988
SARA score	− 0.02 ± 0.01	− 1.53	− 0.04, − 0.01	0.128
INAS count	− 0.03 ± 0.03	− 0.94	− 0.09, 0.03	0.349
Diagnosis	0.16 ± 0.14	1.18	− 0.11, 0.44	0.238
Cluster	1.12 ± 0.13	8.39	0.86, 1.38	< **0.001**
**Visuospatial function**				
Age (years)	− 0.01 ± 0.01	− 0.78	− 0.02, 0.01	0.439
Education (years)	0.05 ± 0.03	1.61	− 0.01, 0.10	0.110
Sex	− 0.35 ± 0.17	− 2.08	− 0.69, − 0.02	**0.039**
Disease duration (years)	− 0.02 ± 0.01	− 2.40	− 0.04, − 0.00	**0.017**
SARA score	− 0.02 ± 0.02	− 1.30	− 0.05, 0.01	0.195
INAS count	0.08 ± 0.05	1.73	− 0.01, 0.17	0.087
Diagnosis	0.09 ± 0.20	0.43	− 0.31, 0.48	0.670
Cluster	1.69 ± 0.19	8.80	1.31, 2.06	< **0.001**
**Neuropsychiatric function**				
Age (years)	0.03 ± 0.01	2.47	0.01, 0.06	**0.014**
Education (years)	− 0.03 ± 0.06	− 0.49	− 0.15, 0.09	0.627
Sex	− 0.07 ± 0.36	− 0.21	− 0.78, 0.63	0.837
Disease duration (years)	− 0.02 ± 0.02	− 0.92	− 0.05, 0.02	0.357
SARA score	− 0.01 ± 0.03	− 0.41	− 0.08, 0.05	0.681
INAS count	− 0.06 ± 0.10	− 0.66	− 0.25, 0.13	0.509
Diagnosis	− 0.72 ± 0.42	− 1.73	− 1.54, 0.10	0.085
Cluster	3.97 ± 0.40	9.90	3.18, 4.76	< **0.001**
**Episodic memory**				
Age (years)	− 0.03 ± 0.01	− 4.07	− 0.04, − 0.02	< **0.001**
Education (years)	0.03 ± 0.03	1.02	− 0.03, 0.09	0.312
Sex	0.34 ± 0.18	1.89	− 0.02, 0.70	0.061
Disease duration (years)	0.01 ± 0.01	1.56	− 0.00, 0.03	0.120
SARA score	0.02 ± 0.02	1.14	− 0.01, 0.05	0.255
INAS count	− 0.09 ± 0.05	− 1.76	− 0.18, 0.01	0.081
Diagnosis	− 0.13 ± 0.21	− 0.62	− 0.54, 0.29	0.537
Cluster	0.96 ± 0.20	4.75	0.56, 1.36	< **0.001**

A total of 166 patients were included in the multiple linear regression analysis

*SARA* Scale for the Assessment and Rating of Ataxia, *INAS* Inventory of Non-Ataxia Signs, *SE* standard error, *CI* confidence interval

## Discussion

In this study, two cognitive clusters were identified within a large, heterogeneous sample of patients with various cerebellar disorders using data-driven cluster analysis. Cluster 1 represented a group of patients with severe cognitive deficits. Cluster 2 represented a group of patients with no or only mild cognitive impairment. Cognitive performance of patients in cluster 2 overlapped substantially with that of healthy controls. Statistically significant differences between patients in cluster 2 and healthy controls, however, were present within the executive, linguistic, and neuropsychiatric domain indicating that subtle deficits exist even in mildly impaired patients. The finding of a cognitively severely and a mildly impaired cluster of patients agrees with previous findings. Other studies which have used the CCAS-S in different languages have shown that some patients are severely impaired, failing three or more test items of the CCAS-S, while other patients only are mildly impaired, failing one or no item [7, 9, 12, 66, 70].

The patients in cluster 1 demonstrated considerably lower *z*-scores on all cognitive and the neuropsychiatric domain compared to the healthy control group. Statistically significant differences were also present within the executive, linguistic and neuropsychiatric domain, but not in the visuospatial domain between patients in cluster 2 and healthy controls. Regarding the percentage of patients that exhibited impairments in a certain CCAS domain, over 90% of patients in cluster 1 and 29% in cluster 2 exhibited impairments in executive and/or linguistic functions, marking the most affected cognitive domains, followed by the neuropsychiatric domain (cluster 1: 81%, cluster 2: 28%). Visuospatial functions were less frequently affected (cluster 1: 44%, cluster 2: 8%).

The finding of more severe impairments in executive and linguistic compared to visuospatial functions is in line with a meta-analysis dealing with the assessment of the CCAS. That meta-analysis revealed the largest effect sizes on tests of executive and linguistic functions compared to tests of visuospatial function. The test that differentiated best between patients and controls was the Stroop test. The Stroop test is designed to assess the inhibition of an overlearned response as well as cognitive flexibility. Inhibition and cognitive flexibility are subdomains of executive functions [1]. Likewise, the studies which have utilized the CCAS-S in different languages (including the German, Dutch, Spanish, and Portuguese version of the CCAS-S) have found the most prominent differences between cerebellar patients and healthy controls on the test items that assess the executive and linguistic domain [12, 36, 51, 66].

The finding of a high rate of abnormal test results within the neuropsychiatric domain is also in line with the literature. Previous studies have reported high rates of neuropsychiatric symptoms in cerebellar disorders ranging from 51 to 77% [33, 35] which is within the same range as the portion of patients with neuropsychiatric deficits in our sample, especially for the severely impaired patients in cluster 1 (cluster 1: 81%, cluster 2: 28%). A recent study found that nearly all patients with cerebellar disorders (95%) show neuropsychiatric symptoms when considering subclinical diagnostic criteria [32]. The latter indicates that the CCAS-S might need further improvements regarding the neuropsychiatric domain. As yet, evaluation is made semi-objectively by the examiner based on a checklist for neuropsychiatric symptoms.

Other promising tests of neuropsychiatric functions, not yet included in the CCAS-S are tests of social cognition, i.e., tests of mental processes required to understand, generate, and regulate social behavior [4, 72]. It has been shown that the cerebellum is critically involved in processes related to social cognition [73–75]. One test that captures the domain of social cognition would be the *Reading the Mind in the Eyes Test*, which measures the ability to interpret emotions and thoughts from facial expressions, particularly focusing on the eyes [3]. Another test of social cognition is the *Picture Sequencing Test*, which requires the participant to arrange cards depicting (social) actions of persons in a meaningful sequential manner [76]. This computer-based test is also less prone to rater-based biases compared to the CCAS-S. Moreover, the *Emotion Attribution Test* [8] and the *Faux Pas Test* [14], which assess the ability to interpret emotions and to understand others’ mental states, could be helpful to diagnose neuropsychiatric symptoms and deficits within the domain of social cognition. Both tests have been shown to sensitively detect such impairments in cerebellar patients [25].

In addition to the exploration of the cognitive profile within the clusters, demographic and clinical variables were compared between the clusters. We found that patients in cluster 1 were on average older and less educated than those in cluster 2 supporting the previous finding that age and education affect test results of the CCAS-S [51, 59, 66]. Likewise, the approach by Thieme et al. [69] using a correction formula (controlling for sex, age, and education effects) matched the current results better than the approach by Hoche et al. [20] (see Figs. S1a and S2a).

The SARA score showed no significant contribution to cognitive/neuropsychiatric functions in the regression models. This finding may be explained by the functional topography of the cerebellum. The cerebellum is functionally divided into motor and non-motor areas. The two main motor representations are located in the anterior cerebellar lobe (lobules I–V), with some extension into lobule VI and in lobule VIII in the posterior cerebellar lobe. Three non-motor representations have been described in the cortex of the posterolateral cerebellar hemispheres (lobules VI-Crus I and ii; lobules Crus II-VIIB; lobules IX-X) [19]. Likewise, the dentate nucleus consists of a motor area in the rostro-dorsal parts and a non-motor area in the ventro-caudal parts of the nucleus [46, 61]. Lesions and degeneration may affect cognitive and motor functions differently in individual patients, potentially explaining our results of a dissociation between motor and cognitive performance. However, the patients in cluster 1 exhibited more severe ataxia symptoms compared to those in cluster 2, suggesting that individuals with severe ataxia, likely at advanced stages of the disease, also tend to have significant degeneration in cognitive areas. This aligns with previous MRI findings showing that cerebellar degeneration affects both areas due to a shared pathophysiology in patients with spinocerebellar ataxias [24, 44, 45]. In contrast, former studies examining specific hereditary ataxias (e.g., SCA2 or SCA3), have found a correlation between the SARA score and CCAS-S performance [36, 51]. One explanation for the discrepancy between our findings and the previous findings could be that our sample was heterogeneous. The pattern of atrophy is known to differ between disease entities [10, 53, 58]. In SCA2 and SCA3, both cerebellar motor and cognitive areas are similarly affected [50]. In other ataxias, cerebellar motor and cognitive areas might be differently affected [18, 47]. For example, in SCA48 an early involvement of cerebellar cognitive areas is usual, and cognitive symptoms often precede ataxia [18]. Another explanation for the discrepancy of our results in comparison with the results of Rodríguez-Labrada et al. [51] and Maas et al. [36] could be that the cognitive deficits in SCA2 and SCA3 patients go beyond a CCAS. Both SCA2 and SCA3 are known to involve extracerebellar structures, such as the basal ganglia or the cerebral cortex [30, 31, 45, 50]. Although, the CCAS-S is designed to detect the cerebellar cognitive profile and the SARA is designed to capture ataxia severity, it is likely that extracerebellar pathologies contribute both to a poorer CCAS-S score and a higher SARA score. Therefore, the ataxias which regularly involve a substantial amount of extracerebellar structures might be more prone to show correlations between ataxia rating scores and the CCAS-S score.

The extent of extracerebellar involvement, as indicated by the INAS count, influenced the classification of patients into the clusters, with individuals in cluster 1 showing more extracerebellar involvement compared to those in cluster 2. However, the classification based on the specific (genetic) entity (e.g. SCA3, SCA6, FRDA SAOA, MSA-C, etc.) did not reveal this relationship, challenging previous research where patients with the same entities were grouped and compared with other entities [29, 60]. This indicates that the individual degree of extracerebellar involvement might be a better predictor of cognitive perfomance in cerebellar patients than the overall classification based on entities.

Cluster assignment remained a significant predictor of G-CCAS-S performance even after accounting for all other demographic and clinical variables. The latter indicates that factors which were not captured in this study likely contribute to cognitive performance in patients. The individual pattern of atrophy/lesions within cerebellar motor and cognitive areas or in other brain areas could be one of these factors. We did not perform MRI-based lesion-symptom mapping which could have answered this question and is a limitation of this study. In the future, the identified clusters should be further characterized using MRI and laboratory biomarkers.

The discriminative abilities of the G-CCAS-S as measured by the AUC were much better in cluster 1 compared to cluster 2. In cluster 1, the AUC was 0.99 for the total sum raw score and 0.94 for the number of failed items compared to 0.67 and 0.60 respectively in cluster 2. Hence, while the diagnostic accuracy was close to ideal (an AUC of 1.0 would indicate an ideal diagnostic accuracy) in cluster 1, the chance level (of an AUC = 0.5) in discriminating patients from controls was only scarcely exceeded in cluster 2. Also, the diagnostic criteria by Hoche et al. (CCAS possible/probable/definite: cluster 1: 0/3/97% vs. cluster 2: 26/26/30%) and the method by Thieme and colleagues using a correction formula controlling for sex, age and education effects (abnormal test: cluster 1: 97% vs. cluster 2: 62%) showed a similar pattern. Sensitivity (that is: the portion of patients correctly identified as patients/all patients in the sample) was close to 100% in cluster 1 while a substantial portion of patients was not identified as patients in cluster 2 (e.g. 18% false negative results according to the method of Hoche et al. [20] and 38% according to Thieme et al.[66]). Taken together, the current results indicate that the diagnostic accuracy of the G-CCAS-S is nearly perfect for severely affected patients, while it has only limited utility for mildly affected individuals. A recent study involving individuals with pre-symptomatic SCA1 and SCA3 carriers found similar results. The pre-symptomatic mutation carriers did not differ from controls regarding the overall CCAS-S scores or in any of the single test items [59]. Future research should focus more on mildly affected patients and pre-symptomatic mutation carriers since more sensitive and selective tests are needed for this subgroup. As mentioned above tests of social cognition or the Stroop test are promising candidates. Moreover, the addition of more test items that capture executive and linguistic functions (like word fluency tests) might help since these were also significantly affected even in the mildly impaired cluster 2 patients.

Although, this study was able to identify and characterize two distinct cognitive clusters within a large population of cerebellar patients, the study has some limitations. First, the patient cohort tended towards older individuals with a lower level of education. Therefore, the results might not be fully transferable to younger patients and those with a higher level of education. In addition, there is a significant heterogeneity within the patient group in terms of disease entity, ataxia severity and the degree of extracerebellar involvement, which also may limit the transferability to other populations. Also, we did not include pre-symptomatic mutation carriers of hereditary ataxias which might be the focus of future research considering upcoming treatment options (e.g. antisense oligonucleotides). Moreover, this study only considered the impact of some demographic and clinical characteristics on CCAS-S performance, maybe overlooking other variables. For example, fatigue and depression have been shown to impact cognitive functions in cerebellar disorders [39, 40]. Also, the inclusion of MRI and molecular/biochemical biomarkers should be considered to examine possible relationships between atrophy patterns or specific mutations and cognitive performance. Future research should incorporate these variables. Additionally, upcoming studies should focus on more homogeneous populations with patients and pre-symptomatic mutation carriers of specific ataxia subtypes.

## Conclusions

This study investigated cognitive performance in a large sample of cerebellar patients using cluster analysis. This data-driven approach allowed us to identify two cognitive clusters (subgroups). We found that executive, linguistic, and neuropsychiatric functions are the most affected domains and that these domains are even impaired in only mildly affected individuals. The G-CCAS-S in its current form, however, is of limited use in mildly affected patients because of the high overlap with the test results of healthy individuals. Future research needs to focus on the development of more sensitive and selective cognitive screening tests for this subgroup of patients, e.g., by including tests of social cognition and/or adding test items that capture executive and linguistic functions.

## Supplementary Information

Below is the link to the electronic supplementary material.

Supplementary file1 (DOCX 862 KB)

Supplementary file2 (DOCX 62 KB)

## Data Availability

Data underlying the statistics, and the figures is available from the corresponding author upon request.

## References

[1] Ahmadian N, van Baarsen K, van Zandvoort M, Robe P (2019) The cerebellar cognitive affective syndrome—a meta-analysis. Cerebellum (London, England) 18:941–950. 10.1007/s12311-019-01060-2 31392563 10.1007/s12311-019-01060-2 PMC6761084

[2] Aita SL, Beach JD, Taylor SE, Borgogna NC, Harrell MN, Hill BD (2019) Executive, language, or both? An examination of the construct validity of verbal fluency measures. Appl Neuropsychol Adult 26:441–451. 10.1590/s1980-57642011dn0501000610.1080/23279095.2018.1439830 29513079 10.1080/23279095.2018.1439830

[3] Baron-Cohen S, Wheelwright S, Hill J, Raste Y, Plumb I (2001) The “Reading the Mind in the Eyes” test revised version: a study with normal adults, and adults with Asperger syndrome or high-functioning autism. J Child Psychol Psychiatry 42:241–251. 10.1111/1469-7610.00715 11280420

[4] Beer JS, Ochsner KN (2006) Social cognition: a multi level analysis. Brain Res 1079:98–105. 10.1016/j.brainres.2006.01.002 16513097 10.1016/j.brainres.2006.01.002

[5] Benassi M, Garofalo S, Ambrosini F, Sant’Angelo RP, Raggini R, De Paoli G, Ravani C, Giovagnoli S, Orsoni M, Piraccini G (2020) Using two-step cluster analysis and latent class cluster analysis to classify the cognitive heterogeneity of cross-diagnostic psychiatric inpatients. Front Psychol 11:1085. 10.3389/fpsyg.2020.01085 32587546 10.3389/fpsyg.2020.01085 PMC7299079

[6] Cattell RB (1943) The description of personality: basic traits resolved into clusters. Psychol Sci Public Interest 38(4):476–506

[7] Chirino-Pérez A, Marrufo-Meléndez O, Muñoz-López J, Hernandez-Castillo C, Ramirez-Garcia G, Díaz R, Nuñez-Orozco L, Fernandez-Ruiz J (2021) Mapping the cerebellar cognitive affective syndrome in patients with chronic cerebellar strokes. Cerebellum (London, England) 21(22):208–218. 10.1007/s12311-021-01290-3 34109552 10.1007/s12311-021-01290-3

[8] Contaldi E, Sensi M, Colucci F, Capone JG, Braccia A, Nocilla MR, Diozzi E, Contini E, Pelizzari AC, Tugnoli V (2023) Electrophysiological and neuropsychological assessment of cognition in spinocerebellar ataxia type 1 patients: a pilot study. Neurol Sci 44:1597–1606. 10.1007/s10072-022-06597-5 36639526 10.1007/s10072-022-06597-5 PMC10102071

[9] Corben LA, Blomfield E, Tai G, Bilal H, Harding IH, Georgiou-Karistianis N, Delatycki MB, Vogel AP (2024) The role of verbal fluency in the cerebellar cognitive affective syndrome scale in Friedreich ataxia. Cerebellum. 10.1007/s12311-024-01694-x 38642239 10.1007/s12311-024-01694-x PMC11489268

[10] Currie S, Hadjivassiliou M, Craven IJ, Wilkinson ID, Griffiths PD, Hoggard N (2013) Magnetic resonance imaging biomarkers in patients with progressive ataxia: current status and future direction. Cerebellum 12:245–266. 10.1007/s12311-012-0405-3 22828959 10.1007/s12311-012-0405-3

[11] D’Agostino RB Sr, Pencina MJ, Massaro JM, Coady S (2013) Cardiovascular disease risk assessment: insights from Framingham. Glob Heart 8:11–23. 10.1590/s1980-57642011dn0501000610.1016/j.gheart.2013.01.001 23750335 10.1016/j.gheart.2013.01.001 PMC3673738

[12] de Oliveira Scott SS, Pedroso JL, Elias VV, Nóbrega PR, Sobreira EST, de Almeida MP, Gama MTD, Massuyama BK, Barsottini OGP, Frota NAF, Braga-Neto P (2022) Translation, cross-cultural adaptation, and validation to Brazilian Portuguese of the cerebellar cognitive affective/Schmahmann Syndrome Scale. Cerebellum 22(22):282–294. 10.1007/s12311-022-01391-7 35305246 10.1007/s12311-022-01391-7

[13] Diallo A, Jacobi H, Cook A, Labrum R, Durr A, Brice A, Charles P, Marelli C, Mariotti C, Nanetti L, Panzeri M, Rakowicz M, Sobanska A, Sulek A, Schmitz-Hübsch T, Schöls L, Hengel H, Melegh B, Filla A, Antenora A, Infante J, Berciano J, van de Warrenburg BP, Timmann D, Boesch S, Pandolfo M, Schulz JB, Bauer P, Giunti P, Kang JS, Klockgether T, Tezenas du Montcel S (2018) Survival in patients with spinocerebellar ataxia types 1, 2, 3, and 6 (EUROSCA): a longitudinal cohort study. Lancet Neurol 17:327–334. 10.1016/s1474-4422(18)30042-5 29553382 10.1016/S1474-4422(18)30042-5

[14] Dogan I, Tinnemann E, Romanzetti S, Mirzazade S, Costa AS, Werner CJ, Heim S, Fedosov K, Schulz S, Timmann D, Giordano IA, Klockgether T, Schulz JB, Reetz K (2016) Cognition in Friedreich’s ataxia: a behavioral and multimodal imaging study. Ann Clin Transl Neurol 3:572–587. 10.1002/acn3.315 27606341 10.1002/acn3.315 PMC4999591

[15] Erdlenbruch F, Timmann D, Thieme A (2024) Clinical cerebellar neuroscience: ataxias—cognitive and affective symptoms in focal cerebellar lesions. Curr Opin Behav Sci 55:101331. 10.1016/j.cobeha.2023.101331

[16] Faletti R, Battisti G, Discalzi A, Grognardi ML, Martinello S, Oderda M, Gontero P, Bergamasco L, Cassinis MC, Fonio P (2016) Can DW-MRI, with its ADC values, be a reliable predictor of biopsy outcome in patients with suspected prostate cancer? Abdom Radiol 41:926–933. 10.1590/s1980-57642011dn0501000610.1007/s00261-015-0574-x 10.1007/s00261-015-0574-x 27193791

[17] Forgy E (1965) Cluster analysis of multivariate data: efficiency versus interpretability of classifications. Biometrics 21:768–780

[18] Genis D, Ortega-Cubero S, San Nicolás H, Corral J, Gardenyes J, de Jorge L, López E, Campos B, Lorenzo E, Tonda R, Beltran S, Negre M, Obón M, Beltran B, Fàbregas L, Alemany B, Márquez F, Ramió-Torrentà L, Gich J, Volpini V, Pastor P (2018) Heterozygous STUB1 mutation causes familial ataxia with cognitive affective syndrome (SCA48). Neurology 91:e1988–e1998. 10.1212/wnl.0000000000006550 30381368 10.1212/WNL.0000000000006550

[19] Guell X, Gabrieli JDE, Schmahmann JD (2018) Triple representation of language, working memory, social and emotion processing in the cerebellum: convergent evidence from task and seed-based resting-state fMRI analyses in a single large cohort. Neuroimage 172:437–449. 10.1016/j.neuroimage.2018.01.082 29408539 10.1016/j.neuroimage.2018.01.082 PMC5910233

[20] Hoche F, Guell X, Vangel M, Sherman J, Schmahmann J (2018) The cerebellar cognitive affective/Schmahmann syndrome scale. Brain J Neurol 141:248–270. 10.1093/brain/awx317 10.1093/brain/awx317 PMC5837248 29206893

[21] Hopkins B, SKELLAM JG, (1954) A new method for determining the type of distribution of plant individuals. Ann Bot 18:213–227. 10.1093/oxfordjournals.aob.a083391

[22] Jacobi H, Rakowicz M, Rola R, Fancellu R, Mariotti C, Charles P, Dürr A, Küper M, Timmann D, Linnemann C, Schöls L, Kaut O, Schaub C, Filla A, Baliko L, Melegh B, Kang JS, Giunti P, van de Warrenburg BP, Fimmers R, Klockgether T (2013) Inventory of Non-Ataxia Signs (INAS): validation of a new clinical assessment instrument. Cerebellum 12:418–428. 10.1007/s12311-012-0421-3 23090211 10.1007/s12311-012-0421-3

[23] Jolliffe IT, Cadima J (2016) Principal component analysis: a review and recent developments. Philos Trans Roy Soc A Math Phys Eng Sci 374:20150202. 10.1098/rsta.2015.0202 10.1098/rsta.2015.0202 PMC4792409 26953178

[24] Jung BC, Choi SI, Du AX, Cuzzocreo JL, Ying HS, Landman BA, Perlman SL, Baloh RW, Zee DS, Toga AW, Prince JL, Ying SH (2012) MRI shows a region-specific pattern of atrophy in spinocerebellar ataxia type 2. Cerebellum 11:272–279. 10.1007/s12311-011-0308-8 21850525 10.1007/s12311-011-0308-8 PMC3785794

[25] Karamazovova S, Matuskova V, Svecova N, Vyhnalek M (2023) Social cognition in degenerative cerebellar ataxias. Curr Opin Behav Sci 54:101313. 10.1016/j.cobeha.2023.101313

[26] Karantonis JA, Rossell SL, Carruthers SP, Sumner P, Hughes M, Green MJ, Pantelis C, Burdick KE, Cropley V, Van Rheenen TE (2020) Cognitive validation of cross-diagnostic cognitive subgroups on the schizophrenia-bipolar spectrum. J Affect Disord 266:710–721. 10.1007/s12311-011-0308-810.1016/j.jad.2020.01.123 32056949 10.1016/j.jad.2020.01.123

[27] Kassambara A, Mundt F (2020) Factoextra: extract and visualize the results of multivariate data analyses. R Packag Version 107. https://CRANR-project.org/package=factoextra

[28] King M, Hernandez-Castillo CR, Poldrack RA, Ivry RB, Diedrichsen J (2019) Functional boundaries in the human cerebellum revealed by a multi-domain task battery. Nat Neurosci 22:1371–1378. 10.1038/s41593-019-0436-x 31285616 10.1038/s41593-019-0436-x PMC8312478

[29] Klinke I, Minnerop M, Schmitz-Hübsch T, Hendriks M, Klockgether T, Wüllner U, Helmstaedter C (2010) Neuropsychological features of patients with spinocerebellar ataxia (SCA) types 1, 2, 3, and 6. Cerebellum 9:433–442. 10.1007/s12311-010-0183-8 20502998 10.1007/s12311-010-0183-8 PMC2949561

[30] Klockgether T (2018) Chapter 14—Sporadic adult-onset ataxia. In: Manto M, Huisman TAGM (eds) Handbook of clinical neurology. Elsevier, Amsterdam, pp 217–225 10.1016/B978-0-444-64189-2.00014-7 29891060

[31] Klockgether T, Mariotti C, Paulson HL (2019) Spinocerebellar ataxia. Nat Rev Dis Primers 5:24. 10.1038/s41572-019-0074-3 30975995 10.1038/s41572-019-0074-3

[32] Kronemer SI, Slapik MB, Pietrowski JR, Margron MJ, Morgan OP, Bakker CC, Rosenthal LS, Onyike CU, Marvel CL (2021) Neuropsychiatric symptoms as a reliable phenomenology of cerebellar ataxia. Cerebellum 20:141–150. 10.1007/s12311-020-01195-7 33000380 10.1007/s12311-020-01195-7 PMC9487161

[33] Leroi I, O’Hearn E, Marsh L, Lyketsos CG, Rosenblatt A, Ross CA, Brandt J, Margolis RL (2002) Psychopathology in patients with degenerative cerebellar diseases: a comparison to Huntington’s disease. Am J Psychiatry 159:1306–1314. 10.1176/appi.ajp.159.8.1306 12153822 10.1176/appi.ajp.159.8.1306

[34] Lindsay E, Storey E (2017) Cognitive changes in the spinocerebellar ataxias due to expanded polyglutamine tracts: a survey of the literature. Brain Sci. 10.3390/brainsci7070083 28708110 10.3390/brainsci7070083 PMC5532596

[35] Liszewski CM, O’Hearn E, Leroi I, Gourley L, Ross CA, Margolis RL (2004) Cognitive impairment and psychiatric symptoms in 133 patients with diseases associated with cerebellar degeneration. J Neuropsychiatry Clin Neurosci 16:109–112. 10.1176/jnp.16.1.109 14990766 10.1176/jnp.16.1.109

[36] Maas R, Killaars S, van de Warrenburg B, Schutter D (2021) The cerebellar cognitive affective syndrome scale reveals early neuropsychological deficits in SCA3 patients. J Neurol 268(269):3456–3466. 10.1007/s00415-021-10516-7 33743045 10.1007/s00415-021-10516-7 PMC8357713

[37] Maechler M, Rousseeuw P, Struyf A, Hubert M, Hornik K (2023) Cluster: cluster analysis basics and extensions. R package version 2.1.6—For new features, see the 'NEWS' and the 'Changelog' file in the package source). https://CRAN.R-project.org/package=cluster

[38] Malcolm A, Pikoos T, Castle DJ, Rossell SL (2021) Cross-diagnostic cognitive heterogeneity in body dysmorphic disorder and obsessive-compulsive disorder. J Behav Ther Exp Psychiatry 73:101674. 10.1016/j.jbtep.2021.101674 34242980 10.1016/j.jbtep.2021.101674

[39] Martinez ARM, Nunes MB, Faber I, D’Abreu A, Lopes-Cendes Í, França MC (2017) Fatigue and its associated factors in spinocerebellar ataxia type 3/Machado–Joseph disease. Cerebellum 16:118–121. 10.1007/s12311-016-0775-z 27021342 10.1007/s12311-016-0775-z

[40] Mastammanavar VS, Kamble N, Yadav R, Netravathi M, Jain S, Kumar K, Pal PK (2020) Non-motor symptoms in patients with autosomal dominant spinocerebellar ataxia. Acta Neurol Scand 142:368–376. 10.1111/ane.13318 32677041 10.1111/ane.13318

[41] Miller GA, Chapman JP (2001) Misunderstanding analysis of covariance. J Abnorm Psychol 110:40–48. 10.1037/0021-843x.110.1.40 11261398 10.1037//0021-843x.110.1.40

[42] Naeije G, Rai M, Allaerts N, Sjogard M, De Tiège X, Pandolfo M (2020) Cerebellar cognitive disorder parallels cerebellar motor symptoms in Friedreich ataxia. Ann Clin Transl Neurol 7:1050–1054. 10.1002/acn3.51079 32510804 10.1002/acn3.51079 PMC7317641

[43] Naeije G, Schulz JB, Corben LA (2022) The cognitive profile of Friedreich ataxia: a systematic review and meta-analysis. BMC Neurol 22:97. 10.1186/s12883-022-02615-3 35300598 10.1186/s12883-022-02615-3 PMC8928653

[44] Olivito G, Cercignani M, Lupo M, Iacobacci C, Clausi S, Romano S, Masciullo M, Molinari M, Bozzali M, Leggio M (2017) Neural substrates of motor and cognitive dysfunctions in SCA2 patients: a network based statistics analysis. Neuroimage Clin 14:719–725. 10.1016/j.nicl.2017.03.009 28393013 10.1016/j.nicl.2017.03.009 PMC5377430

[45] Olivito G, Siciliano L, Clausi S, Lupo M, Romano S, Masciullo M, Molinari M, Cercignani M, Bozzali M, Leggio M (2020) Functional changes of mentalizing network in SCA2 patients: novel insights into understanding the social cerebellum. Cerebellum 19:235–242. 10.1007/s12311-019-01081-x 31925668 10.1007/s12311-019-01081-x

[46] Palesi F, Ferrante M, Gaviraghi M, Misiti A, Savini G, Lascialfari A, D’Angelo E, Gandini Wheeler-Kingshott CAM (2021) Motor and higher-order functions topography of the human dentate nuclei identified with tractography and clustering methods. Hum Brain Mapp 42:4348–4361. 10.1002/hbm.25551 34087040 10.1002/hbm.25551 PMC8356999

[47] Palvadeau R, Kaya-Güleç ZE, Şimşir G, Vural A, Öztop-Çakmak Ö, Genç G, Aygün MS, Falay O, Başak AN, Ertan S (2020) Cerebellar cognitive-affective syndrome preceding ataxia associated with complex extrapyramidal features in a Turkish SCA48 family. Neurogenetics 21:51–58. 10.1007/s10048-019-00595-0 31741143 10.1007/s10048-019-00595-0

[48] Pelleg D, Moore A (1999) Accelerating exact k-means algorithms with geometric reasoning. In: Proceedings of the fifth ACM SIGKDD international conference on knowledge discovery and data mining. Association for Computing Machinery, San Diego, pp 277–281

[49] Ranavolo A, Serrao M, Varrecchia T, Casali C, Filla A, Roca A, Silvetti A, Marcotulli C, Rondinone BM, Iavicoli S, Draicchio F (2019) The working life of people with degenerative cerebellar ataxia. Cerebellum 18:910–921. 10.1007/s12311-019-01065-x 31468336 10.1007/s12311-019-01065-x

[50] Robertson JW, Adanyeguh I, Bender B, Boesch S, Brunetti A, Cocozza S, Coutinho L, Deistung A, Diciotti S, Dogan I, Durr A, Fernandez-Ruiz J, Göricke SL, Grisoli M, Han S, Mariotti C, Marzi C, Mascalchi M, Mochel F, Nachbauer W, Nanetti L, Nigri A, Ono SE, Onyike CU, Prince JL, Reetz K, Romanzetti S, Saccà F, Synofzik M, Ghizoni Teive HA, Thomopoulos SI, Thompson PM, Timmann D, Ying SH, Harding IH, Hernandez-Castillo CR (2024) The pattern and staging of brain atrophy in spinocerebellar ataxia type 2 (SCA2): MRI volumetrics from ENIGMA-ataxia. bioRxiv. 10.1101/2024.09.16.613281

[51] Rodríguez-Labrada R, Batista-Izquierdo A, González-Melix Z, Reynado-Cejas L, Vázquez-Mojena Y, Sanz Y, Canales-Ochoa N, González-Zaldívar Y, Dogan I, Reetz K, Velázquez-Pérez L (2021) Cognitive decline is closely associated with ataxia severity in spinocerebellar ataxia type 2: a validation study of the Schmahmann Syndrome Scale. Cerebellum (London, England) 21(23):391–403. 10.1007/s12311-021-01305-z 34313938 10.1007/s12311-021-01305-z

[52] Rousseeuw PJ (1987) Silhouettes: a graphical aid to the interpretation and validation of cluster analysis. J Comput Appl Math 20:53–65. 10.1016/0377-0427(87)90125-7

[53] Rüb U, Schöls L, Paulson H, Auburger G, Kermer P, Jen JC, Seidel K, Korf HW, Deller T (2013) Clinical features, neurogenetics and neuropathology of the polyglutamine spinocerebellar ataxias type 1, 2, 3, 6 and 7. Prog Neurobiol 104:38–66. 10.1016/j.pneurobio.2013.01.001 23438480 10.1016/j.pneurobio.2013.01.001

[54] Schmahmann J, Sherman J (1998) The cerebellar cognitive affective syndrome. Brain 121(Pt 4):561–579. 10.1093/brain/121.4.561 9577385 10.1093/brain/121.4.561

[55] Schmahmann JD, Weilburg JB, Sherman JC (2007) The neuropsychiatry of the cerebellum—insights from the clinic. Cerebellum 6:254–267. 10.1080/14734220701490995 17786822 10.1080/14734220701490995

[56] Schmitz-Hübsch T, Coudert M, Giunti P, Globas C, Baliko L, Fancellu R, Mariotti C, Filla A, Rakowicz M, Charles P, Ribai P, Szymanski S, Infante J, van de Warrenburg BP, Dürr A, Timmann D, Boesch S, Rola R, Depondt C, Schöls L, Zdzienicka E, Kang JS, Ratzka S, Kremer B, Schulz JB, Klopstock T, Melegh B, du Montcel ST, Klockgether T (2010) Self-rated health status in spinocerebellar ataxia–results from a European multicenter study. Mov Disord 25:587–595. 10.1002/mds.22740 20175183 10.1002/mds.22740

[57] Schmitz-Hübsch T, du Montcel ST, Baliko L, Berciano J, Boesch S, Depondt C, Giunti P, Globas C, Infante J, Kang J-S, Kremer B, Mariotti C, Melegh B, Pandolfo M, Rakowicz M, Ribai P, Rola R, Schöls L, Szymanski S, van de Warrenburg BP, Dürr A, Klockgether T (2006) Scale for the assessment and rating of ataxia. Neurology 66:1717–1720. 10.1212/01.wnl.0000219042.60538.92 16769946 10.1212/01.wnl.0000219042.60538.92

[58] Seidel K, Siswanto S, Brunt ER, den Dunnen W, Korf HW, Rüb U (2012) Brain pathology of spinocerebellar ataxias. Acta Neuropathol 124:1–21. 10.1007/s00401-012-1000-x 22684686 10.1007/s00401-012-1000-x

[59] Selvadurai LP, Perlman SL, Ashizawa T, Wilmot GR, Onyike CU, Rosenthal LS, Shakkottai VG, Paulson HL, Subramony SH, Bushara KO, Kuo SH, Dietiker C, Geschwind MD, Nelson AB, Gomez CM, Opal P, Zesiewicz TA, Hawkins T, Yacoubian TA, Nopoulos PC, Sha SJ, Morrison PE, Figueroa KP, Pulst SM, Schmahmann JD (2024) The cerebellar cognitive affective/Schmahmann Syndrome Scale in spinocerebellar ataxias. Cerebellum 23(24):1411–1425. 10.1007/s12311-023-01651-0 38165578 10.1007/s12311-023-01651-0 PMC11217149

[60] Sokolovsky N, Cook A, Hunt H, Giunti P, Cipolotti L (2010) A preliminary characterisation of cognition and social cognition in spinocerebellar ataxia types 2, 1, and 7. Behav Neurol 23:395045. 10.1155/2010/395045 10.3233/BEN-2010-0270 PMC5434399 20714058

[61] Steele CJ, Anwander A, Bazin PL, Trampel R, Schaefer A, Turner R, Ramnani N, Villringer A (2016) Human cerebellar sub-millimeter diffusion imaging reveals the motor and non-motor topography of the dentate nucleus. Cereb Cortex. 10.1093/cercor/bhw258 27600851 10.1093/cercor/bhw258

[62] Stephen C, Balkwill D, James P, Haxton E, Sassower K, Schmahmann J, Eichler F, Lewis R (2020) Quantitative oculomotor and nonmotor assessments in late-onset GM2 gangliosidosis. Neurology 94:e705–e717. 10.1212/wnl.0000000000008959 31964693 10.1212/WNL.0000000000008959 PMC7176300

[63] Stoodley C, MacMore J, Makris N, Sherman J, Schmahmann J (2016) Location of lesion determines motor vs. cognitive consequences in patients with cerebellar stroke. NeuroImage Clin 12:765–775. 10.1016/j.nicl.2016.10.013 27812503 10.1016/j.nicl.2016.10.013 PMC5079414

[64] Team R (2020) RStudio: integrated development for R. RStudio, PBC, Boston. http://www.rstudio.com/

[65] Tedesco A, Chiricozzi F, Clausi S, Lupo M, Molinari M, Leggio M (2011) The cerebellar cognitive profile. Brain 134:3672–3686. 10.1093/brain/awr266 22036960 10.1093/brain/awr266

[66] Thieme A, Faber J, Sulzer P, Reetz K, Dogan I, Barkhoff M, Krahe J, Jacobi H, Aktories J, Minnerop M, Elben S, van der Veen R, Müller J, Batsikadze G, Konczak J, Synofzik M, Roeske S, Timmann D (2022) The CCAS-scale in hereditary ataxias: helpful on the group level, particularly in SCA3, but limited in individual patients. J Neurol 269(268):4363–4374. 10.1007/s00415-022-11071-5 35364683 10.1007/s00415-022-11071-5 PMC9293809

[67] Thieme A, Roeske S, Faber J, Sulzer P, Minnerop M, Elben S, Jacobi H, Reetz K, Dogan I, Barkhoff M, Konczak J, Wondzinski E, Siebler M, Mueller O, Sure U, Schmahmann J, Klockgether T, Synofzik M, Timmann D (2020) Validation of a German version of the Cerebellar Cognitive Affective/Schmahmann Syndrome Scale: preliminary version and study protocol. Neurol Res Pract 2:39. 10.1186/s42466-020-00071-3 33324939 10.1186/s42466-020-00071-3 PMC7650062

[68] Thieme A, Röske S, Faber J, Sulzer P, Minnerop M, Elben S, Reetz K, Dogan I, Barkhoff M, Konczak J, Wondzinski E, Siebler M, Hetze S, Müller O, Sure U, Klockgether T, Synofzik M, Timmann D (2021) Reference values for the Cerebellar Cognitive Affective Syndrome Scale: age and education matter. Brain J Neurol 144:e20. 10.1093/brain/awaa417 10.1093/brain/awaa417 33367632

[69] Thieme A, Rubarth K, Faber J, Sulzer P, Reetz K, Dogan I, Barkhoff M, Krahe J, Jacobi H, Aktories J, Minnerop M, Elben S, Huvermann D, Erdlenbruch F, Van der veen R, Müller J, Batsikadze G, Frank B, Köhrmann M, Wondzinski E, Siebler M, Hetze S, Müller O, Sure U, Konczak J, Klockgether T, Synofzik M, Konietschke F, Röske S, Timmann D (2022) Cerebellar Cognitive Affective/Schmahmann Syndrome Scale: need for adjusted cut-off values. Program No 28008 2022 Neuroscience Meeting Planner San Diego, CA: Society for Neuroscience

[70] van der Giessen RS, Satoer D, Koudstaal PJ (2023) The CODECS study: COgnitive DEficits in Cerebellar Stroke. Brain Cogn 173:106102. 10.1016/j.bandc.2023.106102 37922627 10.1016/j.bandc.2023.106102

[71] Van Der Schouw Y, Verbeek A, Ruijs J (1992) ROC curves for the initial assessment of new diagnostic tests. Fam Pract 9:506–511. 10.1093/fampra/9.4.506 1490547 10.1093/fampra/9.4.506

[72] Van Overwalle F (2009) Social cognition and the brain: a meta-analysis. Hum Brain Mapp 30:829–858. 10.1002/hbm.20547 18381770 10.1002/hbm.20547 PMC6870808

[73] Van Overwalle F, Baetens K, Mariën P, Vandekerckhove M (2014) Social cognition and the cerebellum: a meta-analysis of over 350 fMRI studies. Neuroimage 86:554–572. 10.1016/j.neuroimage.2013.09.033 24076206 10.1016/j.neuroimage.2013.09.033

[74] Van Overwalle F, D’Aes T, Mariën P (2015) Social cognition and the cerebellum: a meta-analytic connectivity analysis. Hum Brain Mapp 36:5137–5154. 10.1002/hbm.23002 26419890 10.1002/hbm.23002 PMC6869534

[75] Van Overwalle F, Mariën P (2016) Functional connectivity between the cerebrum and cerebellum in social cognition: a multi-study analysis. Neuroimage 124:248–255. 10.1016/j.neuroimage.2015.09.001 26348560 10.1016/j.neuroimage.2015.09.001

[76] Van Rheenen TE, Rossell SL (2013) Picture sequencing task performance indicates theory of mind deficit in bipolar disorder. J Affect Disord 151:1132–1134. 10.1016/j.jad.2013.07.009 23916306 10.1016/j.jad.2013.07.009

[77] Wachholz TBO, Yassuda MS (2011) The interpretation of proverbs by elderly with high, medium and low educational level: abstract reasoning as an aspect of executive functions. Dement Neuropsychol 5:31–37. 10.1590/s1980-57642011dn05010006 29213717 10.1590/S1980-57642011DN05010006 PMC5619136

[78] Wickham H (2016) ggplot2: elegant graphics for data analysis. Springer, New York. ISBN 978-3-319-24277-4. https://ggplot2.tidyverse.org

[79] Wickham H, Francois R (2015) dplyr: a grammar of data manipulation. R Package Version 043. http://CRANR-project.org/package=dplyr

[80] Wilke C, Pellerin D, Mengel D, Traschütz A, Danzi MC, Dicaire M-J, Neumann M, Lerche H, Bender B, Houlden H, Group RS, Züchner S, Schöls L, Brais B, Synofzik M (2023) GAA-FGF14 ataxia (SCA27B): phenotypic profile, natural history progression and 4-aminopyridine treatment response. Brain 146:4144–4157. 10.1093/brain/awad157 37165652 10.1093/brain/awad157

[81] Yap KH, Kessels RPC, Azmin S, van de Warrenburg B, Mohamed Ibrahim N (2022) Neurocognitive changes in spinocerebellar ataxia type 3: a systematic review with a narrative design. Cerebellum 21:314–327. 10.1093/brain/awad15710.1007/s12311-021-01282-3 34231180 10.1007/s12311-021-01282-3

[82] Youden WJ (1950) Index for rating diagnostic tests. Cancer 3:32–35 15405679 10.1002/1097-0142(1950)3:1<32::aid-cncr2820030106>3.0.co;2-3

[83] Zubin J (1938) A technique for measuring like-mindedness. J Abnorm Soc Psychol 33(4):508–516. 10.1037/h0055441

