# Supplementary material for: Subtypes of cognitive impairment in cerebellar disease identified by cross-diagnostic cluster-analysis: results from a German multicenter study
Source: J Neurol. 2024 Dec 21;272(1):83. doi: 10.1007/s00415-024-12831-1 (PMC11663179; doi:10.1007/s00415-024-12831-1)
Supplement: Supplementary file 1 — Supplementary file1 (DOCX 862 KB) [file 415_2024_12831_MOESM1_ESM.docx]

**Table S1. Diagnoses of cerebellar patients within the clusters.**

| **Entity** | **Cluster 1** | **Cluster 2** | **All patients** |  |  |
| --- | --- | --- | --- | --- | --- |
| **Total** | **62 (30%)** | **143 (70%)** | **205 (100%)** |  |  |
| **Cerebellar plus disease** |  |  | **118 (100%)** |  |  |
| **Autosomal-dominant hereditary ataxias** |  |  |  |  |  |
| Spinocerebellar ataxia type 1 (SCA1) | 4 (80%) | 1 (20%) | 5 (100%) |  |  |
| Spinocerebellar ataxia type 2 (SCA2) | 2 (29%) | 5 (71%) | 7 (100%) |  |  |
| Spinocerebellar ataxia type 3 (SCA3) | | 8 (18%) | 36 (82%) | 44 (100%) |  |
| Spinocerebellar ataxia type 13 (SCA13) | 1 (100%) | 0 (0%) | 1 (100%) |  |  |
| Spinocerebellar ataxia type 28 (SCA28) | 1 (100%) | 0 (0%) | 1 (100%) |  |  |
| Spinocerebellar ataxia type 35 (SCA35) | 0 (0%) | 1 (100%) | 1 (100%) |  |  |
| Spinocerebellar ataxia type 48 (SCA48) | 1 (100%) | 0 (0%) | 1 (100%) |  |  |
| Autosomal-dominant hereditary ataxia with extracerebellar signs (unknown mutation) | 8 (67%) | 4 (33%) | 12 (100%) |  |  |
| **Autosomal-recessive hereditary ataxias** |  |  |  |  |  |
| Friedreich's ataxia (FRDA) | 3 (14%) | 19 (86%) | 22 (100%) |  |  |
| Cerebellar ataxia, neuropathy and vestibular areflexia syndrome (RFC1-CANVAS) | 1 (33%) | 2 (67%) | 3 (100%) |  |  |
| Polymerase-gamma-related ataxia (POLG) | 1 (55%) | 1 (55%) | 2 (100%) |  |  |
| Autosomal recessive spastic ataxia of Charlevoix-Saguenay (ARSACS) | 0 (0%) | 1 (100%) | 1 (100%) |  |  |
| Spastic paraplegia 7 (SPG7) | 1 (100%) | 0 (0%) | 1 (100%) |  |  |
| Ataxia with oculomotor apraxia type 2 (AOA2) | 0 (0%) | 1 (100%) | 1 (100%) |  |  |
| Boucher-Neuhauser syndrome | 0 (0%) | 1 (100%) | 1 (100%) |  |  |
| Early-onset cerebellar ataxia with episodic worsening and early developmental delay (biallelic ATP1A3 mutation) | 1 (100%) | 0 (0%) | 1 (100%) |  |  |
| Leukoencephalopathy with brain stem and spinal cord involvement and lactate elevation (LBSL) | 1 (100%) | 0 (0%) | 1 (100%) |  |  |
| Spinocerebellar ataxia recessive type 8 (SCAR8) | | | 1 (33%) | 2 (67%) | 3 (100%) |
| Spinocerebellar ataxia recessive type 16 (SCAR16) | | | 0 (0%) | 1 (100%) | 1 (100%) |
| **Sporadic ataxias** |  |  |  |  |  |
| Multisystem atrophy - cerebellar type (MSA-C) | 3 (55%) | 3 (55%) | 6 (100%) |  |  |
| **Acquired ataxias** |  |  |  |  |  |
| Autoimmune ataxia | 0 (0%) | 3 (100%) | 3 (100%) |  |  |
| **Cerebellar pure disease** |  |  | **87** (100%) |  |  |
| **Autosomal-dominant hereditary ataxias** |  |  |  |  |  |
| Spinocerebellar ataxia type 6 (SCA6) | 5 (26%) | 14 (74%) | 19 (100%) |  |  |
| Spinocerebellar ataxia type 8 (SCA8) | 3 (100%) | 0 (0%) | 3 (100%) |  |  |
| Spinocerebellar ataxia type 14 (SCA14) | 3 (25%) | 9 (75%) | 12 (100%) |  |  |
| Spinocerebellar ataxia type 27B (SCA27B) | 3 (38%) | 5 (62%) | 8 (100%) |  |  |
| Episodic ataxia (EA) | 1 (14%) | 6 (86%) | 7 (100%) |  |  |
| Autosomal-dominant hereditary ataxia without extracerebellar signs (unknown mutation) | 6 (75%) | 2 (25%) | 8 (100%) |  |  |
| **Autosomal-recessive hereditary ataxias** |  |  |  |  |  |
| Spinocerebellar ataxia recessive type 10 (SCAR10) | 0 (0%) | 3 (100%) | 3 (100%) |  |  |
| **Sporadic ataxias** |  |  |  |  |  |
| Sporadic adult onset ataxia (SAOA) | 1 (13%) | 7 (87%) | 8 (100%) |  |  |
| **Acquired ataxias** |  |  |  |  |  |
| Right-sided cerebellar stroke (7 PICA; 1 SUCA) | 2 (25%) | 6 (75%) | 8 (100%) |  |  |
| Left-sided cerebellar stroke (8 PICA; 1 SUCA) | 0 (0%) | 9 (100%) | 9 (100%) |  |  |
| Bilateral cerebellar stroke  (1 bilateral PICA; 1 right-sided PICA and left-sided SUCA) | 1 (55%) | 1 (55%) | 2 (100%) |  |  |

**Supplementary material S1. CCAS-S correction formula.**

The correction formula for the number of failed CCAS-S test items was intended to control for the known sex, age and education effects on the test performance. The formula was developed using a Poisson regression model. For model training, 80% of the healthy controls in the validation trial of the German CCAS-S were randomly selected. Subsequent validation was performed on the remaining 20% of the controls and all patients with cerebellar degeneration. The formula is expressed as:

Y = e ^(0.99 - 0.13 * Xsex + 0.01 * Xage - 0.1 * Xedu)^

where x_sex_ is 1 if the subject is female and 0 if male, x_age_ is the subject’s age in years, X_edu_ is the number of years of education, and Y represents the predicted number of failed test items for a healthy individual.

Abnormal cognitive function can be assumed if a patient’s actual number of failed test items is greater than or equal to Y. Conversely, scores below Y indicate normal cognitive function [1].

For example, for a 60-year-old male patient with cerebellar disease and 12 years of education who failed two items on the CCAS-S, the predicted number of failed test items Y would be 1.5. In other words, a healthy control with the same sex, age and level of education should on average fail no more than 1.5 test items. Since the example patient actually failed two test items which exceeds the predicted number of failed test items Y, he is classified to have abnormal cognitive function.

A comparison between the actual number of failed test items, the predicted number of failed test items and the classification as abnormal and normal based on the correction formula by Thieme et al. and the classification by Hoche et al. is shown in supplementary material 2.

**Table S2. Comparison of demographics between healthy controls and patients with cerebellar disorders**

|  | Healthy controls *n* = 200 | Cerebellar patients *n* = 205 | Effect size **^a^** | *P* value |
| --- | --- | --- | --- | --- |
| Age (*y*) | 51.4 ± 18.1 | 53.2 ± 14.6 | 0.04 | 0.519 |
| Education (*y*) | 15.4 ± 2.8 | 14.9 ± 3.2 | 0.11 | 0.054 |
| Male, *n* (%) | 92 (46%) | 115 (56%) | 0.10 | 0.042 |

Statistics: Mann-Whitney *U* tests were used for continuous variables and Chi-squared tests for classification variables. a: The effect size was evaluated by the rank biserial correlation for continuous variables and Cramer's *V* index for categorical variables.

*y* years

**Table S3. Silhouette metrics for each cluster number**

| Cluster number | 2 | 3 | 4 | 5 | 6 | 7 | 8 | 9 |
| --- | --- | --- | --- | --- | --- | --- | --- | --- |
| Silhouette metrics | 0.36 | 0.22 | 0.26 | 0.28 | 0.27 | 0.28 | 0.23 | 0.27 |

**Table S4. Performance of G-CCAS-S in clusters**

|  | Healthy controls  *n* = 200 | Cluster 1 *n* = 62 | Cluster 2 *n* = 143 | Effect size ^a^ | *P* value |
| --- | --- | --- | --- | --- | --- |
| Total sum raw score | 99.6 ± 9.3 | 70.9 ± 9.9 | 94.7 ± 8.9 | 0.53 | < 0.001 ^b, c, d^ |
| Number of failed items | 1.4 ± 1.3 | 5.2 ± 1.9 | 1.7 ± 1.2 | 0.48 | < 0.001 ^b, c^ |
| Semantic fluency | 23.3 ± 3.3 | 15.9 ± 4.2 | 21.3 ± 4.2 | 0.31 | < 0.001 ^b, c, d^ |
| Phonemic fluency | 11.7 ± 3.7 | 6.0 ± 2.8 | 9.9 ± 3.9 | 0.22 | < 0.001 ^b, c, d^ |
| Category switching | 12.1 ± 3.0 | 6.7 ± 3.2 | 11.0 ± 3.5 | 0.24 | < 0.001 ^b, c, d^ |
| Digit span forward | 6.3 ± 1.2 | 5.7 ± 1.2 | 6.2 ± 1.1 | 0.03 | < 0.001 ^b, c^ |
| Digit span backward | 4.4 ± 1.0 | 3.6 ± 1.0 | 4.2 ± 0.9 | 0.07 | < 0.001 ^b, c^ |
| Cube draw and copy | 13.7 ± 2.0 | 10.5 ± 2.8 | 14.1 ± 1.7 | 0.27 | < 0.001 ^b, c^ |
| Verbal recall | 13.1 ± 2.0 | 11.2 ± 3.2 | 13.4 ± 1.8 | 0.10 | < 0.001 ^b, c^ |
| Similarities | 7.6 ± 0.7 | 6.1 ± 1.8 | 7.5 ± 0.8 | 0.22 | < 0.001 ^b, c^ |
| Go/ No-go | 1.5 ± 0.7 | 1.0 ± 0.8 | 1.4 ± 0.7 | 0.04 | < 0.001 ^b, c^ |
| Affect | 5.8 ± 0.4 | 4.2 ± 1.3 | 5.6 ± 0.7 | 0.36 | < 0.001 ^b, c, d^ |

Statistics: One-way analysis of variance (ANOVA) followed by Bonferroni post hoc tests were used for continuous variables. a: *ω*² was used to evaluate the effect size. b: Significant difference between healthy control and cluster 1 (*p* < 0.05). c: Significant difference between cluster 1 and cluster 2 (*p* < 0.05). d: Significant difference between healthy control and cluster 2 (*p* < 0.05).

*G-CCAS-S* German Cerebellar Cognitive Affective Syndrome Scale

**Table S5. Analysis of covariance (ANCOVA)**

| Dependent variable | Effect of education | | Effect of age | | Effect of age and education | |
| --- | --- | --- | --- | --- | --- | --- |
|  | Effect size ^a^ | *P* value | Effect size ^a^ | *P* value | Effect size ^a^ | *P* value |
| Total sum raw score | 0.45 | < 0.001^b, c, d^ | 0.49 | < 0.001 ^b, c, d^ | 0.42 | < 0.001 ^b, c, d^ |
| Number failed items | 0.40 | < 0.001 ^b, c^ | 0.45 | < 0.001 ^b, c^ | 0.38 | < 0.001 ^b, c^ |
|  | | | | | | |
| Semantic fluency | 0.27 | < 0.001 ^b, c, d^ | 0.29 | < 0.001 ^b, c, d^ | 0.26 | < 0.001 ^b, c, d^ |
| Phonemic fluency | 0.20 | < 0.001 ^b, c, d^ | 0.21 | < 0.001 ^b, c, d^ | 0.19 | < 0.001 ^b, c, d^ |
| Category switching | 0.22 | < 0.001 ^b, c, d^ | 0.23 | < 0.001 ^b, c, d^ | 0.21 | < 0.001 ^b, c, d^ |
| Digit span forward | 0.02 | 0.011 ^b^ | 0.03 | 0.002 ^b, c^ | 0.01 | 0.022 ^b^ |
| Digit span backward | 0.04 | < 0.001 ^b, c^ | 0.07 | < 0.001 ^b, c^ | 0.04 | < 0.001 ^b, c^ |
| Cube draw and copy | 0.21 | < 0.001 ^b, c^ | 0.24 | <0.001 ^b, c^ | 0.19 | < 0.001 ^b, c^ |
| Verbal recall | 0.08 | < 0.001 ^b, c^ | 0.07 | <0.001 ^b, c^ | 0.06 | < 0.001 ^b, c^ |
| Similarities | 0.16 | < 0.001 ^b, c^ | 0.20 | <0.001 ^b, c^ | 0.15 | < 0.001 ^b, c^ |
| Go/ No-go | 0.04 | < 0.001 ^b, c^ | 0.03 | <0.001 ^b, c^ | 0.03 | < 0.001 ^b, c^ |
| Affect | 0.34 | < 0.001 ^b, c, d^ | 0.37 | < 0.001 ^b, c, d^ | 0.35 | < 0.001 ^b, c, d^ |
|  | | | | | | |
| Executive function | 0.35 | < 0.001 ^b, c, d^ | 0.40 | < 0.001 ^b, c, d^ | 0.32 | < 0.001 ^b, c, d^ |
| Linguistic function | 0.36 | < 0.001 ^b, c, d^ | 0.39 | < 0.001 ^b, c, d^ | 0.35 | < 0.001 ^b, c, d^ |
| Visuospatial function | 0.21 | < 0.001 ^b, c^ | 0.24 | < 0.001 ^b, c^ | 0.19 | < 0.001 ^b, c^ |
| Neuropsychiatric function | 0.34 | < 0.001 ^b, c, d^ | 0.37 | < 0.001 ^b, c, d^ | 0.35 | < 0.001 ^b, c, d^ |
| Episodic memory | 0.08 | < 0.001 ^b, c^ | 0.07 | < 0.001 ^b, c^ | 0.06 | < 0.001 ^b, c^ |

Statistics: ANCOVA analyses were used to adjust the effect of age and education. a: *ω*² was used to evaluate the effect size. b: Significant difference between healthy control and cluster 1 (*p* < 0.05). c: Significant difference between cluster 1 and cluster 2 (*p* < 0.05). d^:^ Significant difference between healthy control and cluster 2 (*p* < 0.05).

**Table S6. Demographics of cluster 1 and matched control sample**

|  | Healthy controls *n* = 60 | Cerebellar patients *n* = 62 | Effect size **^a^** | *P* value |
| --- | --- | --- | --- | --- |
| Age (*y*) | 54.1 ± 18.2 | 57.4 ± 13.4 | 0.09 | 0.37 |
| Education (*y*) | 14.1 ± 2.9 | 13.2 ± 2.7 | 0.16 | 0.12 |
| Male, *n* (%) | 27 (45%) | 33 (53%) | 0.08 | 0.36 |

Statistics: Mann-Whitney *U* tests were used for continuous variables and Chi-squared tests for classification variables. a: The effect size was evaluated by the rank biserial correlation for continuous variables and Cramer's *V* index for categorical variables.

*y* years

**Table S7. Demographics of cluster 2 and matched control group**

|  | Healthy controls *n* = 140 | Cerebellar patients *n* = 143 | Effect size **^a^** | *P* value |
| --- | --- | --- | --- | --- |
| Age (*y*) | 50.2 ± 18.0 | 51.4 ± 14.7 | 0.01 | 0.85 |
| Education (*y*) | 15.9 ± 2.6 | 15.6 ± 3.1 | 0.09 | 0.20 |
| Male, *n* (%) | 65 (46%) | 82 (57%) | 0.10 | 0.07 |

Statistics: Mann-Whitney *U* tests were used for continuous variables and Chi-squared tests for classification variables. a: The effect size was evaluated by using the rank biserial correlation for continuous variables and Cramer's *V* index for categorical variables.

*y* years

**Table S8. Model fit for linear regression assessing the impact of demographic and clinical variables on cognitive and** **neuropsychiatric domains in cerebellar disorders patients**

| Dependent variable | *F* (8, 157) | *P* value | Adjusted *R*^2^ (%) |
| --- | --- | --- | --- |
| Executive function | 20.01 | <0.001 | 48.0 |
| Linguistic function | 18.14 | <0.001 | 45.4 |
| Visuospatial function | 18.04 | <0.001 | 45.2 |
| Neuropsychiatric function | 14.88 | <0.001 | 40.2 |
| Episodic memory | 8.98 | <0.001 | 27.9 |

The *F* value tests the significance of the entire regression model. The independent variables included age (*y*), level of education (*y*), sex, disease duration (*y*), INAS count and SARA score, diagnosis, and cluster category.

*SARA* Scale for the Assessment and Rating of Ataxia, *INAS* The Inventory of Non-Ataxia Signs, *y* years


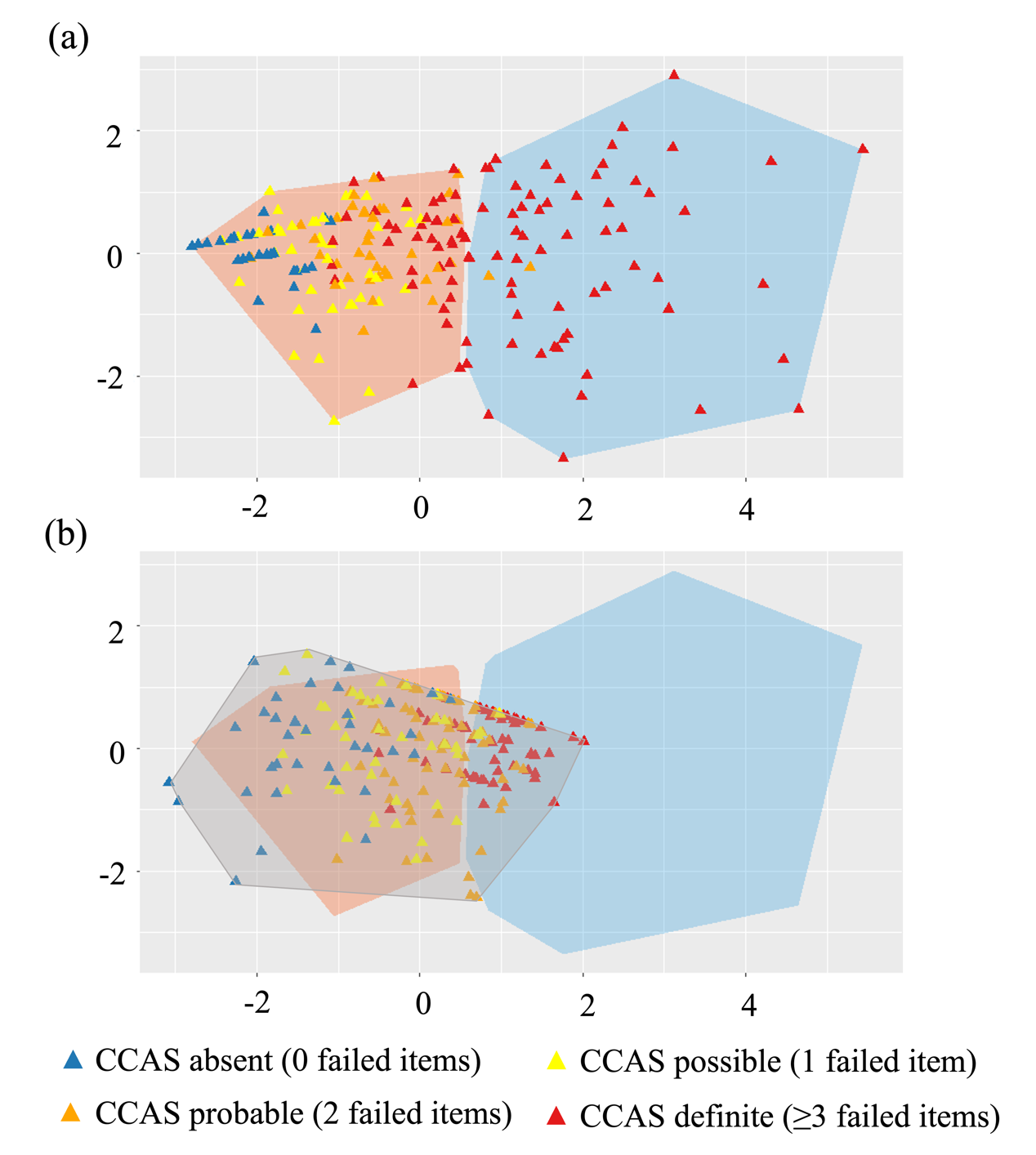


**Fig. S1 Principal component analysis for the cluster analysis.** Categorization of individual participants according to the criteria published by Hoche et al. (2018) in (a) cerebellar patients in cluster 1 (blue shadow) and cluster 2 (red shadow), and (b) healthy controls (grey shadow).

*CCAS* Cerebellar Cognitive Affective Syndrome


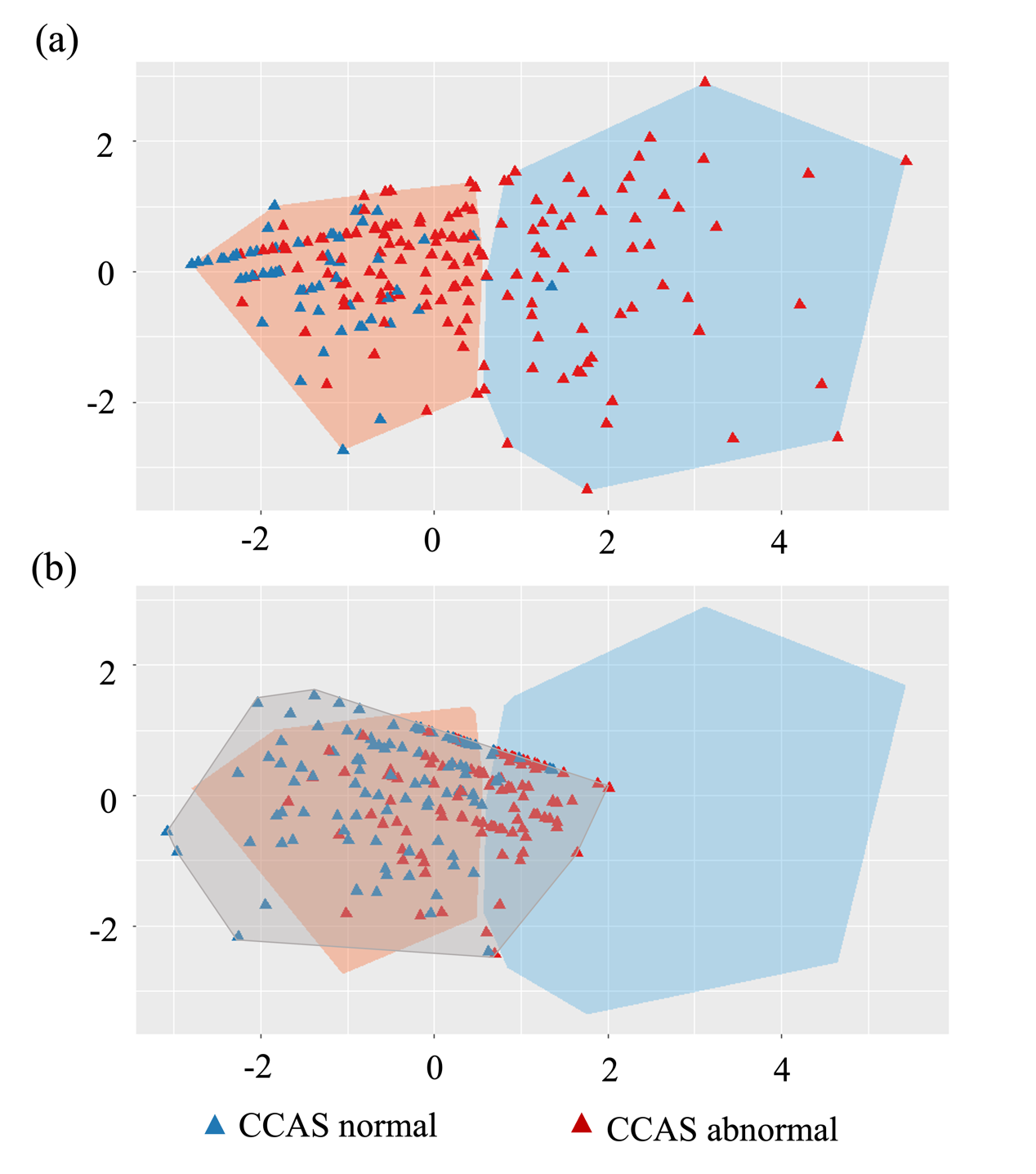


**Fig. S2 Principal component analysis for the cluster analysis.** Categorization of individual participants into *cognitive normal* and *cognitive abnormal* according to the correction formula introduced by Thieme et al. [1] in (a) cerebellar patients in cluster 1 (blue shadow) and cluster 2 (red shadow), and (b) healthy controls (grey shadow).

*CCAS* Cerebellar Cognitive Affective Syndrome

**
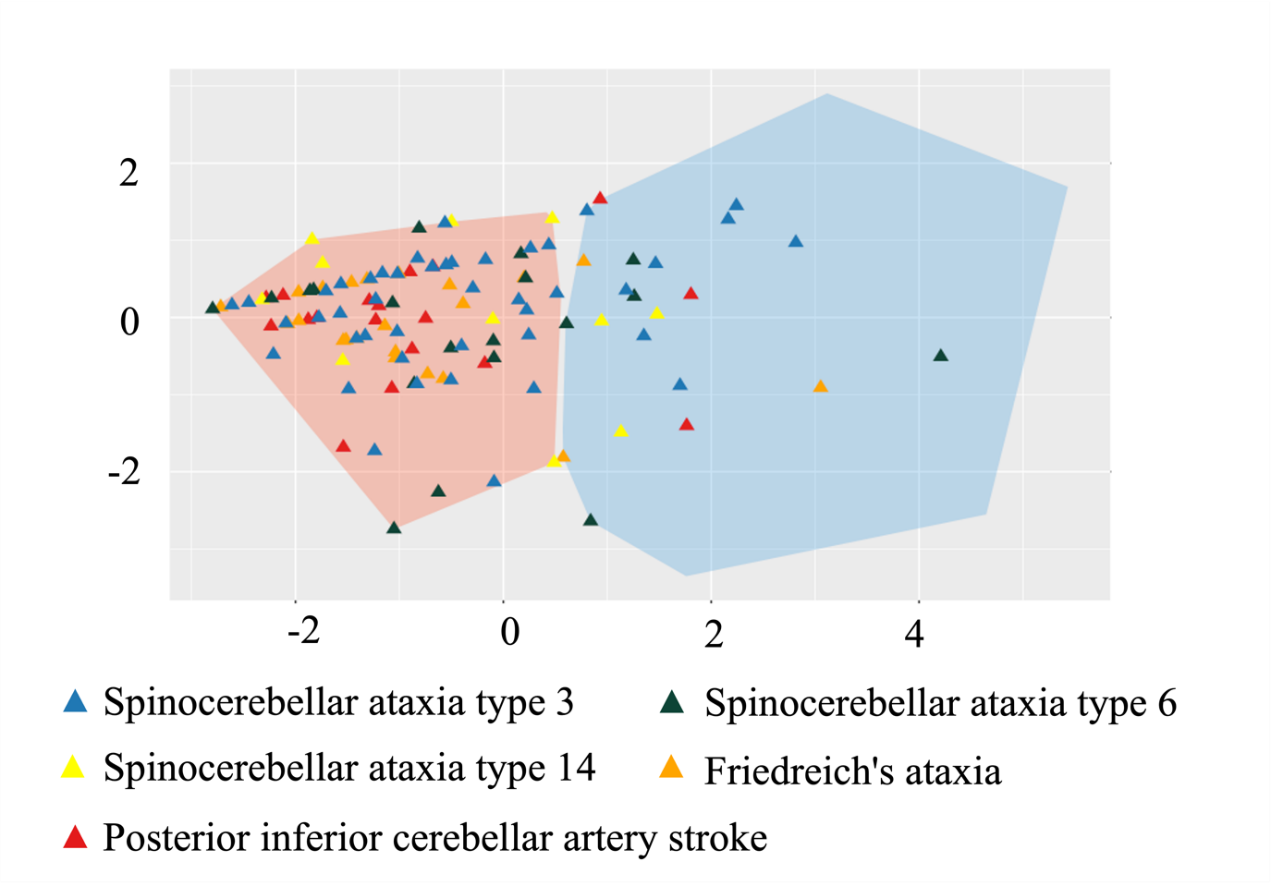
Fig. S3 Distribution of the most common ataxia subtypes in the data set across the clusters.**

**References**

1. Thieme A, Rubarth K, Faber J, Sulzer P, Reetz K, Dogan I, Barkhoff M, Krahe J, Jacobi H, Aktories J, Minnerop M, Elben S, Huvermann D, Erdlenbruch F, Van der veen R, Müller J, Batsikadze G, Frank B, Köhrmann M, Wondzinski E, Siebler M, Hetze S, Müller O, Sure U, Konczak J, Klockgether T, Synofzik M, Konietschke F, Röske S, Timmann D (2022) Cerebellar Cognitive Affective/ Schmahmann Syndrome Scale: Need for adjusted cut-off values. Program No 28008 2022 Neuroscience Meeting Planner San Diego, CA: Society for Neuroscience
