# Supplementary material for: Subtypes of cognitive impairment in cerebellar disease identified by cross-diagnostic cluster-analysis: results from a German multicenter study
Source: J Neurol. 2024 Dec 21;272(1):83. doi: 10.1007/s00415-024-12831-1 (PMC11663179; doi:10.1007/s00415-024-12831-1)
Supplement: Supplementary file 2 — Supplementary file2 (DOCX 62 KB) [file 415_2024_12831_MOESM2_ESM.docx]

**Supplementary material S2. Application of the correction formula: Individual data.**

| **Number** | **Diagnosis** | **Sex** | **Age** | **Level of education** | **Actual number of failed items** | **Predicted number of failed items** | **Classification according to Hoche et al. [1]** | **Classification by use of correction formula [2]** |
| --- | --- | --- | --- | --- | --- | --- | --- | --- |
| 1 | SCA1 | 1 | 58 | 18 | 4.00 | 1.19 | CCAS definite | Abnormal |
| 2 | SCA1 | 1 | 49 | 19 | 5.00 | 0.90 | CCAS definite | Abnormal |
| 3 | SCA1 | 1 | 53 | 13 | 5.00 | 1.76 | CCAS definite | Abnormal |
| 4 | SCA1 | 2 | 47 | 12 | 5.00 | 1.26 | CCAS definite | Abnormal |
| 5 | SCA1 | 1 | 54 | 15 | 5.00 | 1.43 | CCAS definite | Abnormal |
| 6 | SCA2 | 2 | 30 | 21 | 0.00 | 0.40 | CCAS absent | Normal |
| 7 | SCA2 | 1 | 51 | 13 | 3.00 | 1.62 | CCAS definite | Abnormal |
| 8 | SCA2 | 1 | 55 | 14 | 6.00 | 1.60 | CCAS definite | Abnormal |
| 9 | SCA2 | 1 | 63 | 12 | 6.00 | 2.25 | CCAS definite | Abnormal |
| 10 | SCA2 | 2 | 57 | 18 | 4.00 | 0.90 | CCAS definite | Abnormal |
| 11 | SCA2 | 2 | 53 | 16 | 1.00 | 1.04 | CCAS possible | Normal |
| 12 | SCA2 | 2 | 57 | 16 | 1.00 | 1.12 | CCAS possible | Normal |
| 13 | SCA3 | 2 | 70 | 16 | 0.00 | 1.39 | CCAS absent | Normal |
| 14 | SCA3 | 1 | 53 | 19 | 1.00 | 0.98 | CCAS possible | Abnormal |
| 15 | SCA3 | 1 | 51 | 23 | 0.00 | 0.69 | CCAS absent | Normal |
| 16 | SCA3 | 2 | 62 | 12 | 4.00 | 1.70 | CCAS definite | Abnormal |
| 17 | SCA3 | 1 | 40 | 15 | 1.00 | 1.08 | CCAS possible | Normal |
| 18 | SCA3 | 2 | 22 | 12 | 2.00 | 0.76 | CCAS probable | Abnormal |
| 19 | SCA3 | 2 | 50 | 13 | 2.00 | 1.22 | CCAS probable | Abnormal |
| 20 | SCA3 | 1 | 53 | 18 | 2.00 | 1.07 | CCAS probable | Abnormal |
| 21 | SCA3 | 2 | 53 | 16 | 1.00 | 0.99 | CCAS possible | Abnormal |
| 22 | SCA3 | 2 | 23 | 15 | 1.00 | 0.59 | CCAS possible | Abnormal |
| 23 | SCA3 | 2 | 54 | 13 | 0.00 | 1.32 | CCAS absent | Normal |
| 24 | SCA3 | 1 | 63 | 13 | 2.00 | 2.05 | CCAS probable | Normal |
| 25 | SCA3 | 2 | 53 | 16 | 3.00 | 0.99 | CCAS definite | Abnormal |
| 26 | SCA3 | 2 | 45 | 17 | 1.00 | 0.77 | CCAS possible | Abnormal |
| 27 | SCA3 | 2 | 51 | 13 | 1.00 | 1.25 | CCAS possible | Normal |
| 28 | SCA3 | 1 | 23 | 15 | 1.00 | 0.77 | CCAS possible | Abnormal |
| 29 | SCA3 | 1 | 72 | 12 | 4.00 | 2.69 | CCAS definite | Abnormal |
| 30 | SCA3 | 1 | 48 | 18 | 0.00 | 0.97 | CCAS absent | Normal |
| 31 | SCA3 | 2 | 36 | 13 | 3.00 | 0.92 | CCAS definite | Abnormal |
| 32 | SCA3 | 2 | 65 | 13 | 2.00 | 1.65 | CCAS probable | Abnormal |
| 33 | SCA3 | 1 | 50 | 14 | 1.00 | 1.51 | CCAS possible | Normal |
| 34 | SCA3 | 2 | 51 | 17 | 3.00 | 0.87 | CCAS definite | Abnormal |
| 35 | SCA3 | 2 | 54 | 17 | 1.00 | 0.92 | CCAS possible | Abnormal |
| 36 | SCA3 | 2 | 52 | 12 | 7.00 | 1.39 | CCAS definite | Abnormal |
| 37 | SCA3 | 1 | 41 | 17 | 3.00 | 0.92 | CCAS definite | Abnormal |
| 38 | SCA3 | 1 | 48 | 21 | 3.00 | 0.74 | CCAS definite | Abnormal |
| 39 | SCA3 | 1 | 40 | 16 | 4.00 | 0.99 | CCAS definite | Abnormal |
| 40 | SCA3 | 2 | 65 | 13 | 3.00 | 1.65 | CCAS definite | Abnormal |
| 41 | SCA3 | 2 | 53 | 16 | 4.00 | 0.99 | CCAS definite | Abnormal |
| 42 | SCA3 | 2 | 61 | 21 | 3.00 | 0.74 | CCAS definite | Abnormal |
| 43 | SCA3 | 2 | 61 | 13 | 2.00 | 1.59 | CCAS probable | Abnormal |
| 44 | SCA3 | 1 | 51 | 24 | 1.00 | 0.60 | CCAS possible | Abnormal |
| 45 | SCA3 | 2 | 67 | 12 | 1.00 | 1.88 | CCAS possible | Normal |
| 46 | SCA3 | 2 | 67 | 18 | 6.00 | 1.09 | CCAS definite | Abnormal |
| 47 | SCA3 | 2 | 31 | 11 | 4.00 | 1.00 | CCAS definite | Abnormal |
| 48 | SCA3 | 2 | 42 | 14 | 1.00 | 0.95 | CCAS possible | Abnormal |
| 49 | SCA3 | 2 | 48 | 13 | 1.00 | 1.23 | CCAS possible | Normal |
| 50 | SCA3 | 1 | 47 | 22 | 0.00 | 0.66 | CCAS absent | Normal |
| 51 | SCA3 | 1 | 81 | 16 | 2.00 | 2.35 | CCAS probable | Normal |
| 52 | SCA3 | 1 | 35 | 14 | 2.00 | 1.12 | CCAS probable | Abnormal |
| 53 | SCA3 | 1 | 39 | 17 | 3.00 | 0.89 | CCAS definite | Abnormal |
| 54 | SCA3 | 2 | 77 | 14 | 7.00 | 2.00 | CCAS definite | Abnormal |
| 55 | SCA3 | 2 | 58 | 18 | 1.00 | 0.91 | CCAS possible | Abnormal |
| 56 | SCA3 | 1 | 59 | 11 | 4.00 | 2.27 | CCAS definite | Abnormal |
| 57 | SCA13 | 2 | 33 | 17 | 5.00 | 0.61 | CCAS definite | Abnormal |
| 58 | SCA28 | 1 | 58 | 14 | 5.00 | 1.70 | CCAS definite | Abnormal |
| 59 | SCA35 | 1 | 55 | 14 | 0.00 | 1.60 | CCAS absent | Normal |
| 60 | SCA48 | 1 | 69 | 14 | 5.00 | 2.12 | CCAS definite | Abnormal |
| 61 | Autosomal-dominant hereditary ataxia with extracerebellar signs (unknown mutation) | 2 | 59 | 13 | 2.00 | 1.46 | CCAS probable | Abnormal |
| 62 | Autosomal-dominant hereditary ataxia with extracerebellar signs (unknown mutation) | 2 | 48 | 13 | 1.00 | 1.17 | CCAS possible | Normal |
| 63 | Autosomal-dominant hereditary ataxia with extracerebellar signs (unknown mutation) | 1 | 68 | 11 | 3.00 | 2.72 | CCAS definite | Abnormal |
| 64 | Autosomal-dominant hereditary ataxia with extracerebellar signs (unknown mutation) | 2 | 59 | 15 | 2.00 | 1.22 | CCAS probable | Abnormal |
| 65 | Autosomal-dominant hereditary ataxia with extracerebellar signs (unknown mutation) | 2 | 59 | 10 | 9.00 | 1.92 | CCAS definite | Abnormal |
| 66 | Autosomal-dominant hereditary ataxia with extracerebellar signs (unknown mutation) | 1 | 65 | 15 | 4.00 | 1.79 | CCAS definite | Abnormal |
| 67 | Autosomal-dominant hereditary ataxia with extracerebellar signs (unknown mutation) | 2 | 68 | 11 | 7.00 | 2.10 | CCAS definite | Abnormal |
| 68 | Autosomal-dominant hereditary ataxia with extracerebellar signs (unknown mutation) | 2 | 54 | 11 | 7.00 | 1.58 | CCAS definite | Abnormal |
| 69 | Autosomal-dominant hereditary ataxia with extracerebellar signs (unknown mutation) | 1 | 75 | 12 | 6.00 | 2.99 | CCAS definite | Abnormal |
| 70 | Autosomal-dominant hereditary ataxia with extracerebellar signs (unknown mutation) | 1 | 51 | 16 | 1.00 | 1.29 | CCAS possible | Normal |
| 71 | Autosomal-dominant hereditary ataxia with extracerebellar signs (unknown mutation) | 1 | 33 | 10 | 7.00 | 1.48 | CCAS definite | Abnormal |
| 72 | Autosomal-dominant hereditary ataxia with extracerebellar signs (unknown mutation) | 1 | 66 | 12 | 4.00 | 2.39 | CCAS definite | Abnormal |
| 73 | FRDA | 1 | 31 | 20 | 3.00 | 0.60 | CCAS definite | Abnormal |
| 74 | FRDA | 2 | 27 | 19 | 1.00 | 0.45 | CCAS possible | Abnormal |
| 75 | FRDA | 1 | 21 | 13 | 2.00 | 0.93 | CCAS probable | Abnormal |
| 76 | FRDA | 1 | 35 | 16 | 3.00 | 0.90 | CCAS definite | Abnormal |
| 77 | FRDA | 1 | 57 | 20 | 0.00 | 0.97 | CCAS absent | Normal |
| 78 | FRDA | 2 | 52 | 15 | 1.00 | 1.06 | CCAS possible | Normal |
| 79 | FRDA | 1 | 31 | 16 | 0.00 | 0.83 | CCAS absent | Normal |
| 80 | FRDA | 1 | 24 | 13 | 0.00 | 0.94 | CCAS absent | Normal |
| 81 | FRDA | 1 | 25 | 19 | 1.00 | 0.59 | CCAS possible | Abnormal |
| 82 | FRDA | 1 | 29 | 19 | 1.00 | 0.63 | CCAS possible | Abnormal |
| 83 | FRDA | 2 | 28 | 17 | 3.00 | 0.55 | CCAS definite | Abnormal |
| 84 | FRDA | 2 | 56 | 20 | 2.00 | 0.77 | CCAS probable | Abnormal |
| 85 | FRDA | 1 | 31 | 7 | 5.00 | 1.86 | CCAS definite | Abnormal |
| 86 | FRDA | 2 | 28 | 16 | 1.00 | 0.60 | CCAS possible | Abnormal |
| 87 | FRDA | 2 | 54 | 17 | 0.00 | 0.92 | CCAS absent | Normal |
| 88 | FRDA | 1 | 37 | 10 | 4.00 | 1.60 | CCAS definite | Abnormal |
| 89 | FRDA | 2 | 49 | 16 | 0.00 | 0.91 | CCAS absent | Normal |
| 90 | FRDA | 2 | 75 | 12 | 4.00 | 2.20 | CCAS definite | Abnormal |
| 91 | FRDA | 1 | 62 | 18 | 2.00 | 1.28 | CCAS probable | Abnormal |
| 92 | FRDA | 1 | 20 | 13 | 1.00 | 0.87 | CCAS possible | Abnormal |
| 93 | FRDA | 2 | 56 | 13 | 2.00 | 1.38 | CCAS probable | Abnormal |
| 94 | FRDA | 2 | 38 | 18 | 1.00 | 0.61 | CCAS possible | Abnormal |
| 95 | RFC1-CANVAS | 1 | 71 | 18 | 0.00 | 1.54 | CCAS absent | Normal |
| 96 | RFC1-CANVAS | 2 | 61 | 12 | 1.00 | 1.67 | CCAS possible | Normal |
| 97 | RFC1-CANVAS | 1 | 62 | 12 | 5.00 | 2.20 | CCAS definite | Abnormal |
| 98 | POLG | 1 | 27 | 17 | 1.00 | 0.70 | CCAS possible | Abnormal |
| 99 | POLG | 2 | 51 | 14 | 4.00 | 1.14 | CCAS definite | Abnormal |
| 100 | SPG7 | 1 | 47 | 11 | 6.00 | 1.79 | CCAS definite | Abnormal |
| 101 | ARSACS | 1 | 32 | 15 | 1.00 | 0.92 | CCAS possible | Abnormal |
| 102 | AOA2 | 1 | 23 | 18 | 3.00 | 0.62 | CCAS definite | Abnormal |
| 103 | Boucher-Neuhauser syndrome | 1 | 49 | 20 | 4.00 | 0.83 | CCAS definite | Abnormal |
| 104 | Early-onset cerebellar ataxia with episodic worsening and early developmental delay (biallelic ATP1A3 mutation) | 1 | 47 | 10 | 9.00 | 1.95 | CCAS definite | Abnormal |
| 105 | LBSL | 2 | 31 | 16 | 6.00 | 0.64 | CCAS definite | Abnormal |
| 106 | SCAR8 | 1 | 28 | 14 | 2.00 | 0.98 | CCAS probable | Abnormal |
| 107 | SCAR8 | 1 | 29 | 14 | 2.00 | 0.95 | CCAS probable | Abnormal |
| 108 | SCAR8 | 2 | 54 | 9 | 5.00 | 1.90 | CCAS definite | Abnormal |
| 109 | SCAR16 | 1 | 53 | 13 | 3.00 | 1.68 | CCAS definite | Abnormal |
| 110 | MSA-C | 1 | 53 | 16 | 4.00 | 1.28 | CCAS definite | Abnormal |
| 111 | MSA-C | 1 | 62 | 17 | 2.00 | 1.40 | CCAS probable | Abnormal |
| 112 | MSA-C | 2 | 64 | 13 | 1.00 | 1.62 | CCAS possible | Normal |
| 113 | MSA-C | 1 | 66 | 16 | 4.00 | 1.67 | CCAS definite | Abnormal |
| 114 | MSA-C | 1 | 64 | 17 | 9.00 | 1.46 | CCAS definite | Abnormal |
| 115 | MSA-C | 2 | 62 | 14 | 3.00 | 1.48 | CCAS definite | Abnormal |
| 116 | Autoimmune ataxia | 1 | 75 | 18 | 2.00 | 1.67 | CCAS probable | Abnormal |
| 117 | Autoimmune ataxia | 1 | 59 | 16 | 4.00 | 1.45 | CCAS definite | Abnormal |
| 118 | Autoimmune ataxia | 1 | 35 | 21 | 2.00 | 0.60 | CCAS probable | Abnormal |
| 119 | SCA6 | 1 | 33 | 19 | 5.00 | 0.69 | CCAS definite | Abnormal |
| 120 | SCA6 | 1 | 55 | 12 | 2.00 | 1.92 | CCAS probable | Abnormal |
| 121 | SCA6 | 1 | 66 | 19 | 1.00 | 1.27 | CCAS possible | Normal |
| 122 | SCA6 | 1 | 79 | 18 | 3.00 | 1.80 | CCAS definite | Abnormal |
| 123 | SCA6 | 2 | 67 | 13 | 0.00 | 1.72 | CCAS absent | Normal |
| 124 | SCA6 | 1 | 66 | 15 | 1.00 | 1.82 | CCAS possible | Normal |
| 125 | SCA6 | 1 | 59 | 15 | 3.00 | 1.58 | CCAS definite | Abnormal |
| 126 | SCA6 | 1 | 54 | 15 | 3.00 | 1.43 | CCAS definite | Abnormal |
| 127 | SCA6 | 1 | 52 | 15 | 5.00 | 1.38 | CCAS definite | Abnormal |
| 128 | SCA6 | 1 | 59 | 13 | 0.00 | 1.90 | CCAS absent | Normal |
| 129 | SCA6 | 1 | 55 | 23 | 1.00 | 0.71 | CCAS possible | Abnormal |
| 130 | SCA6 | 1 | 49 | 17 | 2.00 | 1.08 | CCAS probable | Abnormal |
| 131 | SCA6 | 1 | 78 | 13 | 3.00 | 2.77 | CCAS definite | Abnormal |
| 132 | SCA6 | 1 | 78 | 18 | 1.00 | 1.77 | CCAS possible | Normal |
| 133 | SCA6 | 2 | 74 | 12 | 3.00 | 2.16 | CCAS definite | Abnormal |
| 134 | SCA6 | 1 | 64 | 9 | 3.00 | 3.00 | CCAS definite | Normal |
| 135 | SCA6 | 2 | 77 | 11 | 10.00 | 2.51 | CCAS definite | Abnormal |
| 136 | SCA6 | 2 | 62 | 12 | 3.00 | 1.70 | CCAS definite | Abnormal |
| 137 | SCA6 | 2 | 55 | 13 | 3.00 | 1.41 | CCAS definite | Abnormal |
| 138 | SCA8 | 1 | 61 | 9 | 8.00 | 2.83 | CCAS definite | Abnormal |
| 139 | SCA8 | 1 | 59 | 13 | 4.00 | 1.90 | CCAS definite | Abnormal |
| 140 | SCA8 | 2 | 62 | 12 | 6.00 | 1.70 | CCAS definite | Abnormal |
| 141 | SCA14 | 1 | 64 | 23 | 0.00 | 0.85 | CCAS absent | Normal |
| 142 | SCA14 | 2 | 38 | 12 | 0.00 | 1.05 | CCAS absent | Normal |
| 143 | SCA14 | 2 | 65 | 12 | 4.00 | 1.80 | CCAS definite | Abnormal |
| 144 | SCA14 | 2 | 72 | 13 | 3.00 | 1.98 | CCAS definite | Abnormal |
| 145 | SCA14 | 1 | 40 | 14 | 0.00 | 1.24 | CCAS absent | Normal |
| 146 | SCA14 | 1 | 61 | 14 | 2.00 | 1.80 | CCAS probable | Abnormal |
| 147 | SCA14 | 1 | 71 | 14 | 3.00 | 2.20 | CCAS definite | Abnormal |
| 148 | SCA14 | 2 | 53 | 16 | 1.00 | 1.04 | CCAS possible | Normal |
| 149 | SCA14 | 1 | 58 | 13 | 3.00 | 1.86 | CCAS definite | Abnormal |
| 150 | SCA14 | 2 | 56 | 23 | 1.00 | 0.59 | CCAS possible | Abnormal |
| 151 | SCA14 | 2 | 59 | 14 | 2.00 | 1.40 | CCAS probable | Abnormal |
| 152 | SCA14 | 1 | 54 | 16 | 5.00 | 1.31 | CCAS definite | Abnormal |
| 153 | SCA27B | 1 | 28 | 21 | 2.00 | 0.52 | CCAS probable | Abnormal |
| 154 | SCA27B | 2 | 76 | 12 | 2.00 | 2.25 | CCAS probable | Normal |
| 155 | SCA27B | 2 | 79 | 11 | 5.00 | 2.61 | CCAS definite | Abnormal |
| 156 | SCA27B | 1 | 62 | 15 | 3.00 | 1.68 | CCAS definite | Abnormal |
| 157 | SCA27B | 2 | 61 | 12 | 0.00 | 1.67 | CCAS absent | Normal |
| 158 | SCA27B | 2 | 76 | 12 | 3.00 | 2.25 | CCAS definite | Abnormal |
| 159 | SCA27B | 1 | 37 | 18 | 2.00 | 0.78 | CCAS probable | Abnormal |
| 160 | SCA27B | 1 | 77 | 12 | 6.00 | 2.97 | CCAS definite | Abnormal |
| 161 | EA | 1 | 39 | 19 | 2.00 | 0.74 | CCAS probable | Abnormal |
| 162 | EA | 1 | 59 | 14 | 2.00 | 1.73 | CCAS probable | Abnormal |
| 163 | EA | 2 | 22 | 15 | 2.00 | 0.61 | CCAS probable | Abnormal |
| 164 | EA | 2 | 53 | 14 | 3.00 | 1.19 | CCAS definite | Abnormal |
| 165 | EA | 2 | 58 | 15 | 1.00 | 1.20 | CCAS possible | Normal |
| 166 | EA | 2 | 56 | 13 | 1.00 | 1.38 | CCAS possible | Normal |
| 167 | EA | 1 | 56 | 8 | 2.00 | 2.80 | CCAS probable | Normal |
| 168 | Autosomal-dominant hereditary ataxia without extracerebellar signs (unknown mutation) | 2 | 56 | 18 | 4.00 | 0.88 | CCAS definite | Abnormal |
| 169 | Autosomal-dominant hereditary ataxia without extracerebellar signs (unknown mutation) | 2 | 65 | 12 | 3.00 | 1.80 | CCAS definite | Abnormal |
| 170 | Autosomal-dominant hereditary ataxia without extracerebellar signs (unknown mutation) | 1 | 33 | 12 | 5.00 | 1.23 | CCAS definite | Abnormal |
| 171 | Autosomal-dominant hereditary ataxia without extracerebellar signs (unknown mutation) | 1 | 63 | 17 | 6.00 | 1.43 | CCAS definite | Abnormal |
| 172 | Autosomal-dominant hereditary ataxia without extracerebellar signs (unknown mutation) | 1 | 50 | 20 | 0.00 | 0.84 | CCAS absent | Normal |
| 173 | Autosomal-dominant hereditary ataxia without extracerebellar signs (unknown mutation) | 2 | 63 | 13 | 1.00 | 1.58 | CCAS possible | Normal |
| 174 | Autosomal-dominant hereditary ataxia without extracerebellar signs (unknown mutation) | 2 | 61 | 14 | 7.00 | 1.39 | CCAS definite | Abnormal |
| 175 | Autosomal-dominant hereditary ataxia without extracerebellar signs (unknown mutation) | 1 | 68 | 14 | 8.00 | 2.08 | CCAS definite | Abnormal |
| 176 | SCAR10 | 2 | 32 | 15 | 4.00 | 0.71 | CCAS definite | Abnormal |
| 177 | SCAR10 | 2 | 32 | 13 | 0.00 | 0.85 | CCAS absent | Normal |
| 178 | SCAR10 | 2 | 53 | 14 | 4.00 | 1.19 | CCAS definite | Abnormal |
| 179 | SAOA | 1 | 65 | 13 | 3.00 | 2.08 | CCAS definite | Abnormal |
| 180 | SAOA | 2 | 65 | 13 | 1.00 | 1.65 | CCAS possible | Normal |
| 181 | SAOA | 1 | 59 | 13 | 2.00 | 1.90 | CCAS probable | Abnormal |
| 182 | SAOA | 1 | 57 | 18 | 2.00 | 1.16 | CCAS probable | Abnormal |
| 183 | SAOA | 2 | 80 | 13 | 2.00 | 2.23 | CCAS probable | Normal |
| 184 | SAOA | 2 | 53 | 13 | 3.00 | 1.30 | CCAS definite | Abnormal |
| 185 | SAOA | 1 | 70 | 17 | 4.00 | 1.65 | CCAS definite | Abnormal |
| 186 | SAOA | 2 | 79 | 12 | 9.00 | 2.39 | CCAS definite | Abnormal |
| 187 | Right-sided PICA stroke | 1 | 63 | 14 | 2.00 | 1.88 | CCAS probable | Abnormal |
| 188 | Right-sided PICA stroke | 1 | 68 | 19 | 0.00 | 1.32 | CCAS absent | Normal |
| 189 | Right-sided PICA stroke | 2 | 68 | 8 | 3.00 | 2.75 | CCAS definite | Abnormal |
| 190 | Right-sided PICA stroke | 1 | 50 | 21 | 0.00 | 0.77 | CCAS absent | Normal |
| 191 | Right-sided PICA stroke | 1 | 30 | 18 | 2.00 | 0.68 | CCAS probable | Abnormal |
| 192 | Right-sided PICA stroke | 2 | 57 | 12 | 7.00 | 1.54 | CCAS definite | Abnormal |
| 193 | Right-sided PICA stroke | 1 | 47 | 16 | 0.00 | 1.14 | CCAS absent | Normal |
| 194 | Right-sided SUCA stroke | 1 | 53 | 18 | 1.00 | 1.07 | CCAS possible | Normal |
| 195 | Left-sided PICA stroke | 2 | 51 | 16 | 0.00 | 0.95 | CCAS absent | Normal |
| 196 | Left-sided PICA stroke | 2 | 48 | 11 | 3.00 | 1.40 | CCAS definite | Abnormal |
| 197 | Left-sided PICA stroke | 1 | 65 | 18 | 2.00 | 1.36 | CCAS probable | Abnormal |
| 198 | Left-sided PICA stroke | 1 | 64 | 18 | 2.00 | 1.34 | CCAS probable | Abnormal |
| 199 | Left-sided PICA stroke | 1 | 59 | 17 | 1.00 | 1.38 | CCAS possible | Normal |
| 200 | Left-sided PICA stroke | 1 | 59 | 18 | 2.00 | 1.21 | CCAS probable | Abnormal |
| 201 | Left-sided PICA stroke | 1 | 19 | 13 | 2.00 | 0.89 | CCAS probable | Abnormal |
| 202 | Left-sided PICA stroke | 1 | 56 | 16 | 1.00 | 1.36 | CCAS possible | Normal |
| 203 | Left-sided SUCA stroke | 1 | 69 | 12 | 0.00 | 2.65 | CCAS absent | Normal |
| 204 | Bilateral cerebellar stroke (right PICA, left SUCA) | 1 | 50 | 15 | 1.00 | 1.40 | CCAS possible | Normal |
| 205 | Bilateral cerebellar stroke (bilateral PICA) | 2 | 51 | 14 | 5.00 | 1.10 | CCAS definite | Abnormal |

*CCAS-S* Cerebellar Cognitive Affective Syndrome, *SCA* Spinocerebellar ataxia, *FRDA* Friedreich's ataxia, *RFC1-CANVAS* Cerebellar ataxia, neuropathy and vestibular areflexia syndrome, *POLG* Polymerase-gamma-related ataxia, *ARSACS* Autosomal recessive spastic ataxia of Charlevoix-Saguenay, *SPG7* Spastic paraplegia 7, *AOA2* Ataxia with oculomotor apraxia type 2, *LBSL* Leukoencephalopathy with brain stem and spinal cord involvement and lactate elevation, *SCAR* Spinocerebellar ataxia recessive type, *MSA-C* Multisystem atrophy - cerebellar type, *EA* Episodic ataxia, *SAOA* Sporadic adult onset ataxia, *BA* basilar artery, *PICA* posterior inferior cerebellar artery, *SUCA* superior cerebellar artery.

**References**

1. Hoche F, Guell X, Vangel M, Sherman J, Schmahmann J (2018) The cerebellar cognitive affective/Schmahmann syndrome scale. Brain : a journal of neurology 141:248-270.<https://doi.org/10.1093/brain/awx317>

2. Thieme A, Rubarth K, Faber J, Sulzer P, Reetz K, Dogan I, Barkhoff M, Krahe J, Jacobi H, Aktories J, Minnerop M, Elben S, Huvermann D, Erdlenbruch F, Van der veen R, Müller J, Batsikadze G, Frank B, Köhrmann M, Wondzinski E, Siebler M, Hetze S, Müller O, Sure U, Konczak J, Klockgether T, Synofzik M, Konietschke F, Röske S, Timmann D (2022) Cerebellar Cognitive Affective/ Schmahmann Syndrome Scale: Need for adjusted cut-off values. Program No 28008 2022 Neuroscience Meeting Planner San Diego, CA: Society for Neuroscience
